# Comparing SARS-CoV-2 testing positivity rates and COVID-19 impact among different isolation strategies: a rapid systematic review and a modelling study

**DOI:** 10.1016/j.eclinm.2023.102058

**Published:** 2023-06-22

**Authors:** Ya Gao, Yunli Zhao, Xi Zhang, Jinhui Tian, Gordon Guyatt, Qiukui Hao

**Affiliations:** a Evidence-Based Medicine Center, School of Basic Medical Sciences, Lanzhou University, Lanzhou, China; b Department of Health Research Methods, Evidence, and Impact, McMaster University, Hamilton, ON, Canada; c National Clinical Research Center for Geriatrics, West China Hospital, Sichuan University, Chengdu, China; d Department of Mathematics and Statistics, McMaster University, Hamilton, ON, Canada; e Department of Medicine, McMaster University, Hamilton, ON, Canada; f School of Rehabilitation Science, McMaster University, Hamilton, ON, Canada

**Keywords:** COVID-19, Isolation duration, Transmission, Meta-analysis, Modelling study

## Abstract

**Background:**

The optimal isolation duration for patients with COVID-19 remains unclear. To support an update of World Health Organization (WHO)'s Living Clinical management guidelines for COVID-19 (https://www.who.int/publications/i/item/WHO-2019-nCoV-clinical-2022.2), this rapid systematic review and modelling study addresses the effects of different isolation periods for preventing onward transmission leading to hospitalisation and death among secondary cases.

**Methods:**

We searched the WHO COVID-19 database for studies up to Feb 27, 2023. We included clinical studies of any design with COVID-19 patients confirmed by PCR test or rapid antigen test addressing the impact of any isolation strategy on preventing the spread of COVID-19. There were no restrictions on publication language, publication status, age of patients, severity of COVID-19, variants of SARS-COV-2, comorbidity of patients, isolation location, or co-interventions. We performed random-effects meta-analyses to summarise testing rates of persistent test positivity rates after COVID-19 infection. We performed pre-specified subgroup analyses by symptom status and meta-regression analyses for the proportion of fully vaccinated patients. We developed a model to compare the effects of three isolation strategies on onward transmission leading to hospitalisation and death. The three isolation strategies were (1) 5-day isolation, with no test to release; (2) removal of isolation based on a negative test; and (3) 10-day isolation, with no test to release. The model incorporates estimates of test positivity rates, effective reproduction number, isolation adherence, false negative rate, and hospitalisation rates or case fatality rates. To assess the impact of varying isolation adherence and false negative rates on rapid antigen testing, we conducted some sensitivity analyses. We used the Grading of Recommendations Assessment, Development and Evaluation approach to assess certainty of evidence. The protocol is registered with PROSPERO (CRD42022348626).

**Findings:**

Fifteen studies addressing persistent test positivity rates including 4188 patients proved eligible. Asymptomatic patients (27.1%, 95% CI: 15.8%–40.0%) had a significantly lower rapid antigen test positive rate than symptomatic patients (68.1%, 95% CI: 40.6%–90.3%) on day 5. The rapid antigen test positive rate was 21.5% (95% CI: 0–64.1%; moderate certainty) on day 10. Our modelling study suggested that the risk difference (RD) for asymptomatic patients between 5-day isolation and 10-day isolation in hospitalisations (23 more hospitalisations of secondary cases per 10,000 patients isolated, 95% uncertainty interval (UI) 14 more to 33 more) and mortality (5 more per 10,000 patients, 95% UI 1 to 9 more) of secondary cases proved very small (very low certainty). For symptomatic patients, the potential impact of 5- versus 10-day isolation was much greater in hospitalisations (RD 186 more per 10,000 patients, 95% UI 113 more to 276 more; very low certainty) and mortality (RD 41 more per 10,000 patients, 95% UI 11 more to 73 more; very low certainty). There may be little or no difference between removing isolation based on a negative antigen test and 10-day isolation in the onward transmission leading to hospitalisation or death, but the average isolation period (mean difference −3 days) will be shorter for the removal of isolation based on a negative antigen test (moderate certainty).

**Interpretation:**

5 days versus 10 days of isolation in asymptomatic patients may result in a small amount of onward transmission and negligible hospitalisation and mortality; however, in symptomatic patients, the level of onward transmission is concerning and may lead to high hospitalisation and death rates. The evidence is, however, very uncertain.

**Funding:**

10.13039/100004423WHO.

**Research in context**

**Evidence before this study**

Isolating patients with confirmed COVID-19 and quarantining individuals with a high risk of recent infection remain widely used strategies to prevent the spread of SARS-CoV-2. There are no prior systematic reviews to evaluate effects relevant to decisions regarding protocols for ending COVID-19 isolation. Many modelling studies have, however, evaluated the impact of 5 days of isolation or alternative strategies (e.g. 7 days and 10 days) with or without one negative lateral flow device on secondary infections or additional transmission risk. However, none has focused on the most patient-important outcomes of onward transmission leading to hospitalisation or death. The optimal isolation duration for patients with COVID-19 remains unclear. To address this knowledge gap, we aimed to conduct random-effects meta-analyses, using the WHO COVID-19 database, to summarise testing rates of persistent test positivity rates after COVID-19 infection and use a model to compare the effects of the 5-day isolation and removal of isolation based on a negative antigen test with 10-day isolation on onward transmission leading to hospitalisation and death.

**Added value of this study**

To our knowledge, this is the first systematic review and modelling study to provide estimates of the most patient-important outcomes of onward transmission leading to hospitalisation or death for the 5-day isolation and removal of isolation based on a negative antigen test versus 10-day isolation. This study demonstrates that for symptomatic patients the 5-day isolation may increase onward transmission and thus hospitalisation and mortality of secondary cases compared with the 10-day isolation. For asymptomatic patients, the increase in hospitalisations and death was small. Removal of isolation based on a negative antigen test will probably shorten the average isolation period compared with isolating all patients for 10 days.

**Implications of all the available evidence**

Our study provides evidence that 5-day isolation, compared to 10-day isolation, may result in a small amount of onward transmission and subsequent hospitalisation and mortality in asymptomatic patients; in contrast, in symptomatic patients, the level of onward transmission is concerning and may lead to high hospitalisation and death rates.

## Introduction

Coronavirus disease 2019 (COVID-19) has, as of March 26, 2023, resulted in over 761 million confirmed cases and more than 6.8 million deaths worldwide.^1^ Healthcare setting and community transmissions play an important role in the spread of the disease. Isolating infected patients and quarantining individuals with a high risk of recent infection remain widely used strategies to prevent the spread of severe acute respiratory syndrome coronavirus 2 (SARS-CoV-2).^2^^,^^3^

The World Health Organization (WHO) recommendations, published on June 17 2020^4^ and September 15 2022,^5^ for discharging patients from isolation differ for symptomatic and asymptomatic patients. For symptomatic patients, WHO recommends 10 days after symptom onset plus at least three additional days without symptoms. For asymptomatic patients, WHO recommends 10 days after a positive test for SARS-CoV-2.^4^^,^^5^

Since the COVID-19 outbreak, SARS-CoV-2 variants have changed from the initial outbreak strain to the delta variant and, most recently, the omicron variant. Compared to previous SARS-CoV-2 variants, the omicron variant is both more transmissible and has a shorter incubation period but has lower viral loads at diagnosis and a generally less severe course.^6^, ^7^, ^8^ Isolation or quarantine to limit its spread has high economic, societal, and psychological costs.^9^, ^10^, ^11^ These findings led the WHO to review its recommendations regarding isolation.

There are no prior systematic reviews to evaluate effects relevant to decisions regarding protocols for ending COVID-19 isolation. Many modelling studies have, however, evaluated impact of 5 days of isolation or alternative strategies (e.g. 7 days and 10 days) with or without one negative lateral flow device on secondary infections or additional transmission risk.^12^, ^13^, ^14^, ^15^ However, none has focused on the most patient-important outcomes - onward transmission leading to hospitalisation or death.

To support an update of WHO Living Clinical management guidelines for COVID-19,^5^ we conducted a rapid systematic review and a modelling study. In the review, we evaluated SARS-CoV-2 testing positivity rates after isolation (i.e., 5–14 days) following diagnosis. In the modelling study, we evaluated the impact of 5-day isolation, removal of isolation based on a negative antigen test, and 10-day isolation periods on onward transmission leading to hospitalisation and death.

## Methods

This rapid systematic review adhered to the Cochrane guidance for rapid reviews^16^ and the Preferred Reported Items for Systematic Reviews and Meta-Analyses 2020 (PRISMA 2020) statement.^17^ We registered this rapid systematic review protocol with PROSPERO (CRD42022348626).

We conducted this review following the WHO predefined population, intervention, comparator, and outcome criteria: randomised controlled trials or observational studies that directly compared the impact of 5-day isolation and removal of isolation based on a negative antigen test with the current WHO-recommended isolation period of 10 days for patients with COVID-19 on onward transmission leading to hospitalisation or death. Because we found no direct evidence addressing the question in either randomised trials or observational studies, we included evidence regarding SARS-CoV-2 testing positivity (i.e., viral culture, rapid antigen test, and PCR test) from 5 days after documented infection onward. On the basis of this evidence, and the best evidence regarding a model to estimate onward transmission leading to hospitalisation or death.

### Eligibility criteria

We included clinical studies of any design with patients with COVID-19 confirmed by PCR test or rapid antigen test addressing the impact of any isolation strategy on preventing the spread of COVID-19. There were no restrictions on publication language, publication status (peer-reviewed, in press, or preprint), age of patients, the severity of COVID-19, variants of SARS-COV-2, comorbidity of patients, isolation location, or co-interventions. We excluded studies enrolling people with suspected or probable COVID-19 (over 20% of participants) or contacts with confirmed COVID-19.

### Outcomes

The patient-important outcomes of interest were onward transmission leading to hospitalisation or death. Viral culture positivity, rapid antigen test positivity, and PCR test positivity provided indirect evidence.

### Data sources and searches

With the aid of an expert librarian from the WHO, we searched the WHO COVID-19 database up to February 27, 2023. The WHO COVID-19 database is a comprehensive multilingual source of current literature on the topic, including global literature from over 25 bibliographic and grey literature sources. Appendix 1 presents the details of the search strategy.

### Study selection

We used Covidence (https://covidence.org/) for screening. Two reviewers independently screened titles and abstracts and subsequently the full texts of potentially eligible records. Reviewers resolved disagreements by discussion or, if necessary, by consultation with a third reviewer.

### Data extraction

Using a predesigned form, a reviewer conducted data extraction, and a second reviewer checked for the correctness and completeness of extracted data. Reviewers resolved discrepancies by discussion and, when necessary, with adjudication by a third reviewer. We extracted the following data: study characteristics (first author, study design, publication year, publication status, country, and sample size); patient characteristics (age, sex, severity of COVID-19, symptom status, vaccination status, and SARS-CoV-2 variant of concern); characteristics of isolation (isolation periods, isolation location, and co-interventions); and data on each outcome of interest.

### Risk of bias assessment

To assess the risk of bias in eligible studies regarding test positivity after infection, we used five domains of the Quality In Prognosis Studies (QUIPS) tool^18^: study participation, study attrition, prognostic factor measurement, outcome measurement, and statistical analysis and reporting. A reviewer rated each domain as either low, moderate, or high risk of bias. A second reviewer verified the judgments. Reviewers resolved discrepancies by discussion and, when necessary, with adjudication by a third reviewer. We considered studies were at overall low risk of bias if we judged four or more domains at low risk of bias; studies were at high risk of bias if we judged one or more domains at high risk of bias. We judged the remaining studies were at moderate risk of bias.

### Statistical analysis

#### Systematic review analysis

The protocol included plans to pool hospitalisation and mortality data (PROSPERO: CRD42022348626) but such direct evidence proved unavailable. Eligible studies reported the SARS-CoV-2 virus testing positivity rates following diagnosis. We performed meta-analyses for rapid antigen test, viral culture, and PCR test data, separately. Using R (version 4.1.1, R Foundation for Statistical Computing) we performed meta-analyses to estimate proportions and associated 95% confidence intervals (CIs) using the restricted maximum likelihood (REML) method with the random-effects model.^19^ We used Freeman-Tukey double arcsine transformation to stabilise variances.^20^ We assessed the between-study heterogeneity with a visual inspection of forest plots and the I^2^ statistic. When 10 or more studies were available for an outcome, we planned to assess publication bias using Egger's test.^21^ To ensure the robustness of results, we performed sensitivity analyses only including studies with a low risk of bias.

#### Modelling study

To estimate the impact of different isolation strategies on patient import outcomes (i.e., onward transmission leading to hospitalisation and death), we first performed a modelling study (multiplication equations) and further conducted a more complex microsimulation modelling study as a sensitivity analysis. We modelled the impact of three different isolation strategies for COVID-19: 5-day isolation (that is, patients with COVID-19 are isolated for 5 days, then can end isolation without any further consideration); removal of isolation based on negative antigen test (that is, patients with COVID-19 are isolated and receive a rapid antigen test daily from day 5 to day 9, those who test negative can end isolation while those who test positive continue to isolate until test negative or day 10); 10-day isolation (that is, patients with COVID-19 are isolated for 10 days, then can end isolation without any further consideration). We compared the impact of the 5-day isolation and the removal of isolation based on a negative antigen test with 10-day isolation on onward transmission leading to hospitalisation and death.

We modelled a sample of 10,000 individuals with confirmed COVID-19. To estimate the hospitalisation and death for secondary cases of 5-day isolation and 10-day isolation, we used an effective secondary reproduction number of 0.96 (95% CI 0.72 to 1.2),^22^ a hospitalisation rate of 4.14%,^23^ and a case fatality rate of 0.91%.^1^ To calculate 95% uncertainty intervals (UIs), we considered the 95% CIs of the effective secondary reproduction number and uncertainty intervals of hospitalisation rate (3.35%–4.93%) and fatality rate (0.33%–1.30%).^23^^,^^24^ We made several assumptions in the modelling study. First, we assumed 100% adherence to isolation. Second, we assumed that patients with a positive test (i.e., rapid antigen, viral culture) were infectious and those with a negative test were not. Additionally, we assumed that patients who tested negative would remain negative. To estimate the number of hospitalisation and death for secondary cases, we used multiplication equations (test positivity per 10,000 patients multiplied by the effective reproduction number and the hospitalisation rate or case fatality rate). To assess the impact of varying isolation adherence and false negative rates on rapid antigen testing, we also conducted sensitivity analyses, specifically considering isolation adherence rates of 80% and 50% and false negative rates of 20% and 33%.^25^^,^^26^

Given the assumption that patients with a negative rapid antigen test are non-infectious, onward transmission, hospitalisation, and death using strategies of terminating isolation at the first negative antigen test will be identical to the 10-day isolation strategy. To calculate the average isolation period associated with the strategy of isolation terminated with the first negative test, we used the following equation:

$$Average\;isolation\;period=rapid\;antigen\;test\;positivity\;on\;day\;5\;\times \;5+\;\sum_{k=6}^{9}\left(rapid\;antigen\;test\;positivity\;on\;day\;k-rapid\;antigen\;test\;positivity\;on\;day\;\left(k-1\right)\right)\;\times \;k$$

As a sensitivity analysis, we further performed a more complex microsimulation modelling study using the model developed by Quilty and colleagues,^27^ revised to suit our study purposes. In the complex microsimulation model, using a baseline Ct level of 40, an incubation period of 3.42 days,^28^ a peak Ct value of 22.3 for symptomatic individuals,^29^ viral shedding time for symptomatic infections of 19.7 days (95%CI 17.2 to 22.7), asymptomatic infections of 10.9 days (95% CI 8.3–14.3 days),^30^ and day 5, day 6, and day 10 rapid antigen test positivity or viral culture positivity data from our rapid systematic review, we simulated a viral load trajectory of Ct values over the course of infection for each patient. We assumed that if the Ct value is less than 30, the individual is infectious.^31^ Appendix 2 presents the detailed methods.

### Subgroup and meta-regression analysis

As requested by the WHO guideline panel, we performed pre-specified subgroup analyses by symptom status (asymptomatic versus symptomatic patients with a prior subgroup hypothesis that asymptomatic patients would have a lower positive rate). We performed meta-regression analyses for the proportion of fully vaccinated patients (hypothesising a lower positive rate in studies with a higher proportion of fully vaccinated patients). If at least two studies provided information on a subgroup, we performed within-study subgroup analyses. To assess the credibility of significant subgroup effects, we used a version of the Instrument for assessing the Credibility of Effect Modification Analyses (ICEMAN) tool, originally developed for randomised trials and meta-analysis of randomised trials, modified for the issue of test positivity over the post-infection period.^32^ A finding of moderate or highly credible subgroup effects mandated a focus on subgroup results to inform the WHO panel's recommendations.

### Certainty of evidence

For results of meta-analyses of SARS-CoV-2 testing positivity rates following diagnosis, we used the GRADE (Grading of Recommendations Assessment, Development, and Evaluation) approach for overall prognosis in broad populations^33^ to rate the overall certainty of the evidence for each outcome as ‘‘very low’’, ‘‘low’’, ‘‘moderate’’, or ‘‘high’’. The assessment included five domains: risk of bias,^34^ imprecision,^35^ inconsistency,^36^ indirectness,^37^ and publication bias.^38^ To assess the certainty of the evidence from our modelling study, we used criteria adapted from GRADE guidelines for assessing the certainty of modelled evidence.^39^

### Ethics approval and consent to participate

Not applicable.

### Role of the funding source

The funder had no role in study design, data collection, analysis, and interpretation, or writing of the manuscript and the decision to submit.

## Results

### Systematic review of clinical studies

#### Study identification

**Fig. 1:**
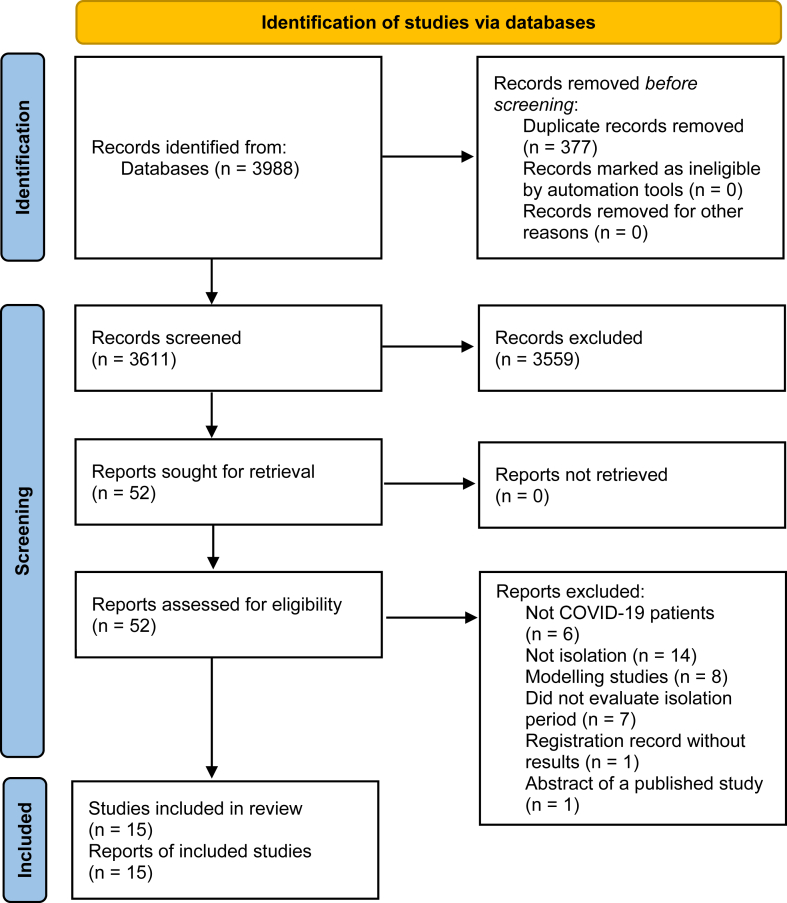
**Flow diagram of study selection**.

The electronic database search identified 3988 records. After screening 3611 titles and abstracts and 52 full texts, 15 studies^40^, ^41^, ^42^, ^43^, ^44^, ^45^, ^46^, ^47^, ^48^, ^49^, ^50^, ^51^, ^52^, ^53^, ^54^ proved eligible (Fig. 1).

#### Characteristics of eligible studies

Table 1 summarises the 15 cohort studies that proved eligible, 13 of which were published in 2022, two were published in 2023, and all addressed COVID-19 positivity from 5 to 14 days after diagnosis. 10 studies were conducted in the United States, two in South Korea, one in Canada, one in Italy, and one in Saudi Arabia. The sample size ranged from 10 to 1023 (a total of 4118) and the proportion of males was from 28.1% to 90.0%. Most studies included patients with omicron SARS-CoV-2 variants, although eight studies did not provide the distribution of omicron variants. Two studies included only asymptomatic patients, two only symptomatic patients, eight both asymptomatic and symptomatic patients, and three did not provide relevant information.

**Table 1:** Characteristics of eligible studies

Study	Publication status	Design	Country	Number of patients	Mean age	Male %	Variant of concern	Severity	Symptom status	Vaccination status	Isolation location	Outcomes
Alshukairi 2022	Peer-reviewed publication	Cohort	Saudi Arabia	480	37.5	36.67	did not report proportion	100% (non-severe)	100% (asymptomatic)	74.58% (fully vaccinated, not boosted), 24.79% (fully vaccinated, boosted), 0.63% (unknown)	NR	RAT positivity
Bouton 2023	Peer-reviewed publication	Cohort	USA	92	22	38.04	81.52% (omicron), 18.48% (delta)	NR	70.65% (symptomatic), 29.35% (asymptomatic)	64.13% (fully vaccinated, not boosted), 35.87% (fully vaccinated, boosted)	NR	Culture positivity
Cosimi 2022	Preprint	Cohort	USA	40	32	42.5	86–99% (omicron)	100% (non-severe)	82.5% (symptomatic), 17.5% (asymptomatic)	10% (fully vaccinated, not boosted), 90% (fully vaccinated, boosted)	NR	RAT positivity; culture positivity
Côté 2022	Peer-reviewed publication	Cohort	Canada	10	61	90	100% (alpha)	100% (severe)	100% (symptomatic)	NR	COVID-19 specific units	Culture positivity
Earnest 2022	Peer-reviewed publication	Cohort	USA	323	18-22^b^	NR	did not report proportion	NR	63.47% (symptomatic), 35.60% (asymptomatic), 0.93% (unknown)	2.79% (one dose), 26.63% (two doses), 68.11% (three doses), 0.61% (four doses), 1.86% (unknown)	NR	RAT positivity
Jang 2022	Peer-reviewed publication	Cohort	South Korea	11	38^a^	45.45	100% (omicron)	100% (non-severe)	100% (symptomatic)	18.18% (fully vaccinated), 81.82% (unvaccinated)	Negative-pressure isolation room	Culture positivity
Jung 2023	Peer-reviewed publication	Cohort	South Korea	32	29.1	28.13	100% (omicron)	100% (non-severe)	62.5% (symptomatic), 37.5% (asymptomatic)	12.5% (fully vaccinated, not boosted), 87.5% (fully vaccinated, boosted)	Single room with or without negative pressure	Culture positivity; PCR test positivity
Landon 2022	Preprint	Cohort	USA	260	NR	NR	did not report proportion	NR	NR	45.77% (fully vaccinated, not boosted), 54.23% (fully vaccinated, boosted)	University hospital setting	RAT positivity
Lefferts 2022	Peer-reviewed publication	Cohort	USA	729	30^a^	47.87	did not report proportion	NR	77.37% (symptomatic), 22.63% (asymptomatic)	74.21% (fully vaccinated), 22.92% (unvaccinated), 2.88% (partially unvaccinated)	NR	RAT positivity
Mack 2022	Peer-reviewed publication	Cohort	USA	173	NR	NR	did not report proportion	NR	NR	100% (fully vaccinated)	NR	PCR test positivity
Nelson 2022	Preprint	Cohort	USA	448	NR	NR	did not report proportion	NR	38.84% (symptomatic), 23.21% (asymptomatic), 29.02% (unknown)	59.15% (fully vaccinated), 2.46% (partially vaccinated), 17.86% (unvaccinated), 11.61% (unknown)	NR	RAT positivity
Sikka 2022	Preprint	Cohort	USA	37	≥18^b^	NR	100% (omicron)	NR	95.59% (symptomatic), 5.41% (asymptomatic)	8.11% (fully vaccinated, not boosted), 21.62% (fully vaccinated, boosted), 70.27% (partially vaccinated)	NR	PCR test positivity
Stingone 2022	Peer-reviewed publication	Cohort	Italy	196	41^a^	82.14	did not report proportion	100% (non-severe)	100% (asymptomatic)	NR	COVID-19 hotel	PCR test positivity
Tsao 2022	Peer-reviewed publication	Cohort	USA	264	20.1	46.97	100% (omicron)	NR	66.0% (symptomatic), 34.0% (asymptomatic)	100% (fully vaccinated)	Isolation housing	RAT positivity
Wagester 2022	Peer-reviewed publication	Cohort	USA	1023	NR	NR	did not report proportion	NR	NR	NR	NR	RAT positivity

Ct, cycle threshold; RAT, rapid antigen test; PCR, polymerase chain reaction; NR, not reported.

a Age is presented as a median.

b Age is presented as a range.

#### Risk of bias

Eleven studies^40^, ^41^, ^42^^,^^44^^,^^46^^,^^48^^,^^50^, ^51^, ^52^, ^53^, ^54^ had a low risk of bias and four^43^^,^^45^^,^^47^^,^^49^ had a moderate risk of bias. Most biases were due to the lack of a clear definition or description of the prognostic factor (symptom status) and poor reporting of the statistical analysis (Appendix 3).

#### Outcomes

**Fig. 2:**
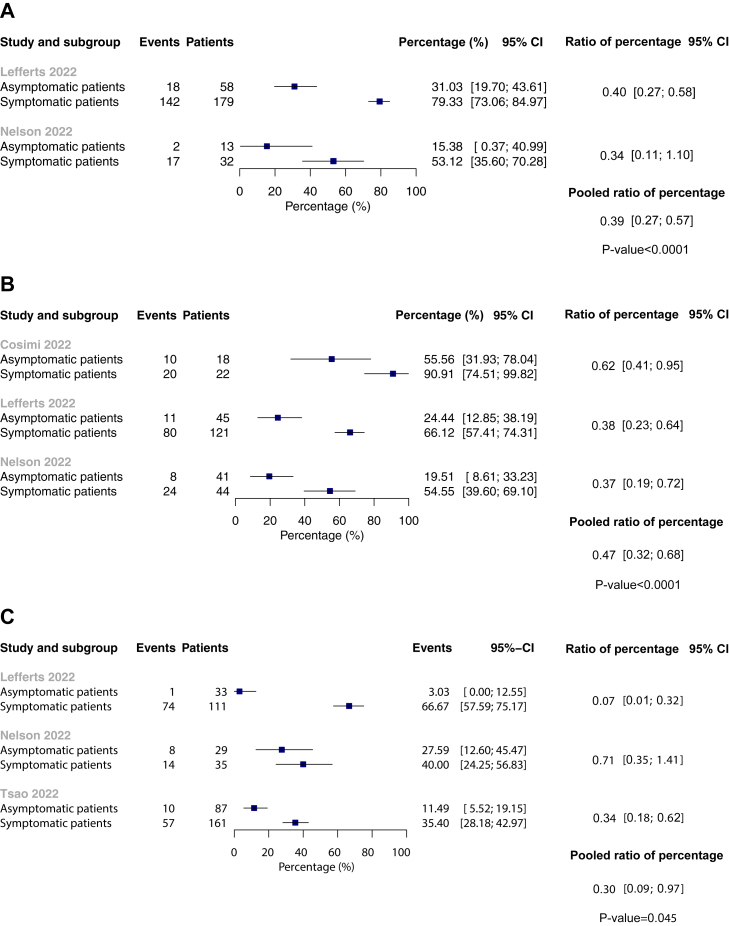
**Within-study subgroup analysis of day 5, day 6, and day 7 rapid antigen test positivity by symptom status**. (A) Day 5; (B) Day 6; (C) Day 7.

**Fig. 3:**
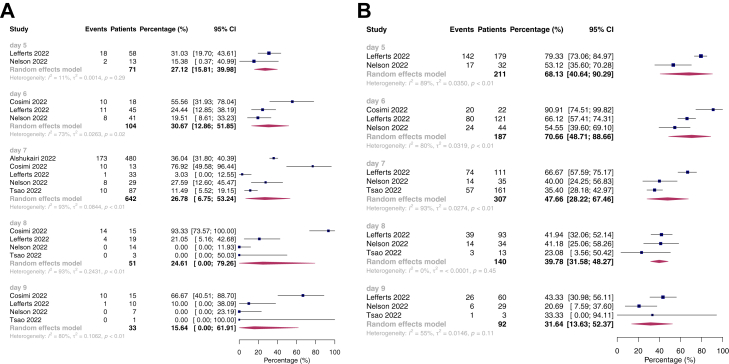
**Pooled percentage of rapid antigen test positivity for asymptomatic and symptomatic patients**. (A) Asymptomatic patients; (B) Symptomatic patients.

Moderate certainty evidence showed the pooled percentage of patients with positive rapid antigen test was 52.3% (95%CI 39.1%–65.3%; 5 studies,^44^^,^^47^^,^^48^^,^^50^^,^^54^ 1690 patients) on isolation day 5; 47.5% (95%CI 28.2%–67.1%; 5 studies,^42^^,^^44^^,^^47^^,^^48^^,^^50^ 691 patients) on day 6; and 21.5% (95%CI 0%–64.1%; 3 studies,^42^^,^^44^^,^^47^ 368 patients) on day 10 (Appendices 4 and 5). Within-study subgroup analyses (Fig. 2 and Appendix 6) showed significant subgroup effects between asymptomatic patients and symptomatic patients on day 5 (pooled ratio of percentage is 0.39, 95% CI: 0.27 to 0.57, P for interaction <0.001), day 6 (pooled ratio of percentage is 0.47, 95% CI: 0.32 to 0.68, P for interaction <0.001), and day 7 (pooled ratio of percentage is 0.30, 95% CI: 0.09 to 0.97, P for interaction = 0.045) rapid antigen test positivity. We judged the credibility of these subgroup effects as moderate (Table 2). Asymptomatic patients had a lower rapid antigen test positive rate than symptomatic patients from day 5 to day 9 (Fig. 3).

**Table 2:** Credibility assessment of subgroup analysis for day 5, 6 and 7 rapid antigen test positivity by symptom status.

ICEMAN item	Assessment for day 5	Assessment for day 6	Assessment for day 7
Is the analysis of effect modification based on comparison within rather than between trials?	Completely within	Completely within	Completely within
For within-trial comparisons, is the effect modification similar from trial to trial?	Definitely similar	Definitely similar	Definitely similar
For between-trial comparisons, is the number of trials large?	Not applicable	Not applicable	Not applicable
Was the direction of effect modification correctly hypothesised a priori?	Definitely yes	Definitely yes	Definitely yes
Does a test for interaction suggest that chance is an unlikely explanation of the apparent effect modification?	Chance an unlikely explanation	Chance an unlikely explanation	Chance a likely explanation
Did the authors test only a small number of effect modifiers or consider the number in their statistical analysis?	Definitely yes	Definitely yes	Definitely yes
Did the authors use a random effects model?	Definitely yes	Definitely yes	Definitely yes
If the effect modifier is a continuous variable, were arbitrary cut points avoided?	Not applicable	Not applicable	Not applicable
Are there any additional considerations that may increase or decrease credibility?	Probably decrease due to small sample size in each subgroup	Probably decrease due to small sample size in each subgroup	Not applicable
How would you rate the overall credibility of the proposed effect modification?	Moderate credibility	Moderate credibility	Moderate credibility

Low certainty evidence revealed that the percentage of patients with positive viral cultures was 37.9% (95%CI 2.8%–82.2%; 2 studies,^45^^,^^46^ 43 patients) on day 5; 28.2% (95%CI 6.0%–57.5%; 4 studies,^41^^,^^42^^,^^45^^,^^46^ 152 patients) on day 6; and 0% (95%CI 0%–15.1%; 1 study,^45^ 11 patients) on day 10 (Appendices 5 and 7). Between-study subgroup analyses indicated that, on day 6, asymptomatic patients had a lower culture-positive rate (22.2% versus 63.5%, P for interaction = 0.05) than symptomatic patients (Appendix 8). However, due to the between-study comparison, small sample size, and chance remaining a likely explanation for findings, we rated the credibility of these subgroup effects as low (Appendix 9).

Meta-regression analyses showed that the proportion of fully vaccinated patients may affect day 10 rapid antigen test positivity and day 6 culture positivity (Appendix 10). However, due to the between-study comparison and the small number of studies, we rated the credibility of these subgroup effects as low (Appendix 11). Sensitivity analyses only including low risk of bias studies showed similar results to primary analyses of all studies (Appendix 12). Appendix 13 presents additional results that include PCR test positivity data. Since we identified fewer than 8 eligible studies for all outcomes, we could not conduct the assessment of publication bias as planned.

### Modelling studies

Our model results suggested, for asymptomatic patients, there may be a small difference between 5-day isolation and 10-day isolation in 28-day hospitalisation (risk difference (RD) 23 more hospitalisations of secondary cases per 10,000 patients isolated, 95% UI 14 more to 33 more) and 90-day mortality (RD 5 more death of secondary cases per 10,000 patients isolated, 95% UI 1 to 9 more). For symptomatic patients, the 5-day isolation may increase hospitalisation (RD 186 more hospitalisations of secondary cases per 10,000 patients isolated, 95% UI 113 more to 276 more) and mortality (RD 41 more deaths of secondary cases per 10,000 patients isolated, 95% UI 11 more to 73 more) compared with the 10-day isolation (Table 3). Appendix 14 presents the GRADE summary of findings for outcomes estimated from positive viral culture data.

**Table 3:** GRADE summary of findings for five-day isolation versus ten-day isolation for outcomes estimated from rapid antigen test data.

Outcome	Absolute effect estimates		Certainty of the evidence	Plain language summary
	Isolation for 5 days	Isolation for 10 days		
**All patients**				
Onward transmission leading to hospitalisation (28 days)	**208** per 10,000	**85** per 10,000	**Very low** Due to certainty of parameters (moderate) in the model and indirectness	Whether isolation of 5 days compared with 10 days would increase onward transmission leading to hospitalisation for secondary cases is very uncertain.
	Difference: **123 more per 10,000** (95% UI 74 more to 182 more)			
Onward transmission leading to death (90 days)	**46** per 10,000	**19** per 10,000	**Very low** Due to certainty of parameters (moderate) in the model and indirectness	Whether isolation of 5 days compared with 10 days would increase onward transmission leading to death for secondary cases is very uncertain.
	Difference: **27 more per 10,000** (95% UI 7 more to 48 more)			
**Asymptomatic patients**				
Onward transmission leading to hospitalisation (28 days)	**108** per 10,000	**85** per 10,000	**Very low** Due to certainty of parameters (moderate) in the model and indirectness	Whether isolation for 5 days compared with 10 days would increase onward transmission leading to hospitalisation of secondary cases is very uncertain.
	Difference: **23 more per 10,000** (95% UI 14 more to 33 more)			
Onward transmission leading to death (90 days)	**24** per 10,000	**19** per 10,000	**Very low** Due to certainty of parameters (moderate) in the model and indirectness	Whether isolation for 5 days compared with 10 days would increase onward transmission leading to death of secondary cases is very uncertain.
	Difference: **5 more per 10,000** (95% UI 1 more to 9 more)			
**Symptomatic patients**				
Onward transmission leading to hospitalisation (28 days)	**271** per 10,000	**85** per 10,000	**Very Low** Due to certainty of parameters (moderate) in the model and indirectness	Whether isolation for 5 days compared with 10 days would increase onward transmission leading to hospitalisation of secondary cases is very uncertain.
	Difference: **186 more per 10,000** (95% UI 113 more to 276 more)			
Onward transmission leading to death (90 days)	**60** per 10,000	**19** per 10,000	**Very Low** Due to certainty of parameters (moderate) in the model and indirectness	Whether isolation for 5 days compared with 10 days would increase onward transmission leading to death of secondary cases is very uncertain.
	Difference: **41 more per 10,000** (95% UI 11 to 73 more)			

UI, uncertainty interval. Model assumptions: we assume that all patients with a negative rapid antigen test are non-infectious and assume if the test is negative, the patients would stay negative status; the isolation adherence is 100%.

There may be little or no difference in the hospitalisation and mortality for secondary cases between removing isolation based on a negative antigen test before 10 days and at the 10-day isolation (very low certainty), but the average isolation period (mean difference −3 days) will probably be shorter for the removal of isolation based on a negative antigen test compared with 10-day isolation (moderate certainty, Table 4). Most estimates from the modelling study are very low certainty.

**Table 4:** GRADE summary of findings for removal of isolation based on a negative antigen test versus ten-day isolation for overall patients.

Outcome	Absolute effect estimates		Certainty of the evidence	Plain language summary
	Removal of isolation based on a negative antigen test	Isolation for 10 days		
Onward transmission leading to hospitalisation (28 days)	**85** per 10,000	**85** per 10,000	**Very Low** Due to certainty of parameters (moderate) in the model and indirectness	Whether removing isolation based on a negative antigen test compared with isolation of 10 days would increase onward transmission leading to hospitalisation of secondary cases is very uncertain.
	Identical			
Onward transmission leading to death (90 days)	**19** per 10,000	**19** per 10,000	**Very Low** Due to certainty of parameters (moderate) in the model and indirectness	Whether removing isolation based on a negative antigen test compared with isolation of 10 days would increase onward transmission leading to death of secondary cases is very uncertain.
	Identical			
Average isolation period	**7 days**	**10 days**	**Moderate** Due to parameters in the model	Removing isolation based on the negative antigen test probably decreases average isolation period compared with isolation for 10 days.
	Mean difference: **3 days lower**			

UI, uncertainty interval. Model assumptions: we assume that all patients with a negative rapid antigen test are non-infectious and assume if the test is negative, the patients would stay negative status; the isolation adherence is 100%.

We conducted sensitivity analyses on the assumptions of the simple model, including different isolation adherence rates and false negative rates. These analyses supported the findings of our primary analyses (Appendices 15 and 16). Additionally, we used a more complex microsimulation model to conduct an additional sensitivity analysis, which provided similar results in onward transmission leading to hospitalisation (RD varied from 183 more to 185 more per 10,000 patients isolated) and death (RD varied from 63 more to 68 more per 10,000 patients isolated) for 5-day isolation versus 10-day isolation (Appendix 17.1). In the complex microsimulation model using assumptions different from our relatively simple model, removal of isolation based on a negative antigen test may also have little difference in hospitalisation and mortality for secondary cases when compared with 10 days of isolation (Appendix 17.2).

## Discussion

In this rapid systematic review of 15 cohort studies including 4118 patients with COVID-19, we found that the pooled percentage of rapid antigen test positivity on day 5 was 52.3% and asymptomatic patients had a lower rapid antigen test positive rate than symptomatic patients (27.1% versus 68.1%) that chance could not easily explain and that met ICEMAN criteria for a moderate credibility subgroup analysis. The percentage of positive cases decreased over the isolation time reaching, for the entire group, 21.5% on day 10.

Our primary modelling study yields very low certainty of evidence on onward transmission leading to hospitalisation or death. It does suggest, however, that for symptomatic patients the 5-day isolation may increase onward transmission and thus hospitalisation and mortality of secondary cases compared with the 10-day isolation by a magnitude most would consider important (Table 3). For asymptomatic patients, the increase in hospitalisations and death may be small enough to be considered unimportant. A second modelling study based on alternative methods showed similar results. Removal of isolation based on a negative antigen test will probably shorten the average isolation period compared with isolating all patients for 10 days.

Our review is limited in that no studies, either randomised or observational, have directly addressed the impact of variable durations of isolation on forward transmission and its important possible consequences on hospitalisation and mortality. We therefore had to address the issue with indirect evidence that informed our modelling study. This modelling study has substantial uncertainties with respect to several parameters including effective reproduction number, hospitalisation rate, and case fatality rate. Some reassurance regarding the credibility of the results comes, however, from similar results in an alternative modelling approach.

In contrast to previous attempts to model the possible consequences of isolation periods, we conducted a systematic review of one key aspect of the relevant indirect evidence: the SARS-CoV-2 virus test positivity from 5 to 14 days after diagnosis. The strengths of this review include a comprehensive search, duplicate assessment of eligibility, independent checking of data abstraction, and appropriate statistical analysis, including the approach to addressing a key subgroup hypothesis: results confirmed the hypothesised markedly greater test positivity rates at 5 days in those with or without symptoms.

Twenty-seven percent of studies in our review were preprints that have yet to undergo peer-review process, and therefore their results may differ from the final published version. However, the likelihood of results changing is low.^55^ We observed substantial heterogeneity across studies in most meta-analyses, although we have performed some analyses to explore the sources of heterogeneity. Factors such as the severity of COVID-19, age, co-intervention, and immunosuppression status may also attribute to the observed heterogeneity. However, we are unable to undertake some pre-specified subgroup analysis to explore the impact of these factors on our outcome due to a lack of available data.

To provide evidence of patient-important outcomes, we further performed modelling studies relying on the results of our review of test positivity studies. Strengths of our model include its transparency. To reflect the currently dominant variant of concern, for input parameters, we used data specific to the Omicron variant when available. Finally, our relatively simple model provided results similar to applying our parameter estimates to a more complex microsimulation model.

The main limitation of the model is uncertainty around key parameters, in particular reproduction number and hospitalisation rate. These include some parameters (e.g., peak Ct value, duration of viral shedding) in which data specific to the Omicron variant were not available, requiring use of data from the overall SARS-COV-2 variants. Owing to limited data, we were unable to estimate outcomes for some subgroups of patients (e.g., vaccination status). These limitations led us to classify the evidence regarding hospitalisation and death as very low certainty. The potential use of repeated lateral flow tests to reduce the duration of quarantine and improve compliance was not investigated in our study. Future studies can consider evaluating the effect of this strategy on patient-important outcomes.

A previous study reported that the transmissibility of asymptomatic cases may be significantly lower than that of symptomatic cases.^56^ Our review found that asymptomatic patients had a lower rate of positive results on rapid antigen tests and fewer cases of onward transmission leading to hospitalisation or death than symptomatic patients. These findings are consistent with the previous study. A randomised, controlled, non-inferiority trial reported that daily lateral flow device testing with 24 h exemption from self-isolation appeared to be non-inferior to standard self-isolation (10 days) in reducing onward transmission of SARS-CoV-2 for contacts of confirmed COVID-19 cases.^57^ Another cluster-randomised, controlled trial reported that daily contact testing of school-based contacts was non-inferior to self-isolation of 10 days for control of COVID-19 transmission.^58^ These findings are consistent with our results on the comparison of removal of isolation based on negative antigen testing and 10-day isolation in confirmed patients with COVID-19.

Healthcare public policy decisions must rely on the best evidence, even if that evidence is of very low quality. Recommendations regarding isolation after COVID-19 diagnosis, because of their major implications for large numbers of people, represent a compelling example. Despite limitations of the evidence regarding the magnitude of hospitalisation resulting from 5 versus 10 days of isolation, because of the moderate credibility of subgroup analysis regarding duration of test positivity related to symptomatic status, there is a high likelihood that shorter isolation will result in appreciably less hospitalisation in the asymptomatic than the symptomatic patients. The WHO panel using the results of our work was therefore able to recommend 5 days of isolation for asymptomatic patients and 10 days for symptomatic patients.

Our studies identify major gaps in the evidence regarding an optimal isolation period. Compelling evidence regarding optimal isolation will require conduct of randomised controlled trials. Future clinical studies could focus on patient-important outcomes (e.g. onward transmission leading to hospitalisation and/or death) and test important subgroup hypotheses such as the variant of virus, disease severity, symptom status, vaccination status, immunosuppression status, and co-interventions.

Our findings show that a 5-day isolation may result in minimal hospitalisation or death as a result of spread of COVID-19 for asymptomatic patients; the same may not be true for symptomatic patients. Removing isolation based on a negative antigen test is likely to shorten the average isolation period, possibly without negative consequences on onward transmission. Providing higher certainty evidence on the impact of alternative isolation strategies will require future studies using robust designs.

## Contributors

YG, GG, and QH conceived and designed the study. YG and YZ screened and selected the articles. YG and YZ extracted the data and assessed the risk of bias. YG, XZ, and QH led the development and analysis of the model. GG supervised the data analyses. YG and QH rated the certainty of evidence. GG provided methodological support. YG, YZ, XZ, GG, JT, and QH interpreted the data. YG and QH drafted the manuscript. All authors reviewed the manuscript and approved the final version of the manuscript. YG and QH accessed and verified the underlying data. All authors had full access to all the data in the study and had final responsibility for the decision to submit for publication.

## Data sharing statement

Data in this systematic review with meta-analysis are extracted from published studies available on the internet. All processed data are presented in this article and the appendix.

## Declaration of interests

We declare no competing interests.
