# Supplementary material for: Comparing SARS-CoV-2 testing positivity rates and COVID-19 impact among different isolation strategies: a rapid systematic review and a modelling study
Source: eClinicalMedicine. 2023 Jun 22;61:102058. doi: 10.1016/j.eclinm.2023.102058 (PMC10285308; doi:10.1016/j.eclinm.2023.102058)
Supplement: Supplementary Appendix [file mmc1.docx]

**Comparing SARS-CoV-2 testing positivity rates and COVID-19 impact among different isolation strategies: a rapid systematic review and a modelling study**

**Contents**

[**Appendix 1: Search strategy of WHO COVID-19 database 4**](#_Toc131609284)

[**Appendix 2: Methods of the complex microsimulation model 5**](#_Toc131609285)

[**Appendix 3. Risk of bias for clinical studies 8**](#_Toc131609286)

[**Appendix 4. Pooled percentage of rapid antigen test positivity for overall patients 9**](#_Toc131609287)

[**4.1. Pooled percentage of rapid antigen test positivity from day 5 to day 8 for overall patients 9**](#_Toc131609288)

[**4.2. Pooled percentage of rapid antigen test positivity from day 9 to day 14 for overall patients 10**](#_Toc131609289)

[**Appendix 5. GRADE assessment for rapid antigen test positivity and viral culture positivity at days 5, 6 and 10 11**](#_Toc131609290)

[**Appendix 6. Within-study subgroup analysis of day 8 and day 9 rapid antigen test positivity by symptom status 12**](#_Toc131609291)

[**6.1. Within-study subgroup analysis of day 8 rapid antigen test positivity by symptom status 12**](#_Toc131609292)

[**6.2. Within-study subgroup analysis of day 9 rapid antigen test positivity by symptom status 13**](#_Toc131609293)

[**Appendix 7. Pooled percentage of viral culture positivity from day 5 to day 14 14**](#_Toc131609294)

[**Appendix 8. Subgroup analysis of day 6 viral culture positivity by symptom status 15**](#_Toc131609295)

[**Appendix 9. Credibility assessment of subgroup effect for day 6 viral culture positivity by symptom status 16**](#_Toc131609296)

[**Appendix 10. Meta-regression analysis by proportion of fully vaccinated patients 18**](#_Toc131609297)

[**10.1. Meta-regression analysis for day 5 rapid antigen test positivity by proportion of fully vaccinated patients 18**](#_Toc131609298)

[**10.2. Meta-regression analysis for day 6 rapid antigen test positivity by proportion of fully vaccinated patients 19**](#_Toc131609299)

[**10.3. Meta-regression analysis for day 7 rapid antigen test positivity by proportion of fully vaccinated patients 20**](#_Toc131609300)

[**10.4. Meta-regression analysis for day 8 rapid antigen test positivity by proportion of fully vaccinated patients 21**](#_Toc131609301)

[**10.5. Meta-regression analysis for day 9 rapid antigen test positivity by proportion of fully vaccinated patients 22**](#_Toc131609302)

[**10.6. Meta-regression analysis for day 10 rapid antigen test positivity by proportion of fully vaccinated patients 23**](#_Toc131609303)

[**10.7. Meta-regression analysis for day 6 viral culture positivity by proportion of fully vaccinated patients 24**](#_Toc131609304)

[**Appendix 11. Credibility assessment of subgroup effects for rapid antigen test positivity and viral culture positivity by proportion of fully vaccinated patients 25**](#_Toc131609305)

[**11.1. Credibility assessment of subgroup analysis for day 10 rapid antigen test positivity by proportion of fully vaccinated patients 25**](#_Toc131609306)

[**11.2. Credibility assessment of subgroup analysis for day 6 viral culture positivity by proportion of fully vaccinated patients 27**](#_Toc131609307)

[**Appendix 12. Sensitivity analyses only including studies with low risk of bias 29**](#_Toc131609308)

[**12.1. Sensitivity analyses for day 5 to day 8 rapid antigen test positivity only including studies with low risk of bias 29**](#_Toc131609309)

[**12.2. Sensitivity analyses for day 9 to day 14 rapid antigen test positivity only including studies with low risk of bias 30**](#_Toc131609310)

[**12.3. Sensitivity analyses for day 5 to day 14 viral culture positivity only including studies with low risk of bias 31**](#_Toc131609311)

[**Appendix 13. Additional results of rapid systematic review 32**](#_Toc131609312)

[**Appendix 14. GRADE summary of findings for five-day isolation versus ten-day isolation for outcomes estimated using positive viral culture data from relatively simple model for overall patients 34**](#_Toc131609313)

[**Appendix 15. Sensitivity analysis of five-day isolation versus ten-day isolation for outcomes estimated using rapid antigen test data from relatively simple model considering different isolation adherence rates and false negative rates (GRADE summary of findings) 35**](#_Toc131609314)

[**15.1. Sensitivity analysis of five-day isolation versus ten-day isolation for outcomes estimated using rapid antigen test data from relatively simple model using an isolation adherence of 80% and a false negative rate of 20% 35**](#_Toc131609315)

[**15.2. Sensitivity analysis of five-day isolation versus ten-day isolation for outcomes estimated using rapid antigen test data from relatively simple model using an isolation adherence of 80% and a false negative rate of 33% 37**](#_Toc131609316)

[**15.3. Sensitivity analysis of five-day isolation versus ten-day isolation for outcomes estimated using rapid antigen test data from relatively simple model using an isolation adherence of 50% and a false negative rate of 20% 38**](#_Toc131609317)

[**15.4. Sensitivity analysis of five-day isolation versus ten-day isolation for outcomes estimated using rapid antigen test data from relatively simple model using an isolation adherence of 50% and a false negative rate of 33% 39**](#_Toc131609318)

[**Appendix 16. Sensitivity analysis for removal of isolation based on a negative antigen test versus ten-day isolation for estimates from relatively simple model for overall patients considering different isolation adherence rates and false negative rates (GRADE summary of findings) 40**](#_Toc131609319)

[**Appendix 17. Sensitivity analysis of estimates from complex microsimulation model for overall patients (GRADE summary of findings) 42**](#_Toc131609320)

[**17.1. Sensitivity analysis of five-day isolation versus ten-day isolation for estimates from complex microsimulation model 42**](#_Toc131609321)

[**17.2. Sensitivity analysis of removal of isolation based on a negative antigen test versus ten-day isolation for estimates from complex microsimulation model 43**](#_Toc131609322)

# Appendix 1: Search strategy of WHO COVID-19 database

| **#** | **Search** |
| --- | --- |
| 1 | ("infective duration"~5 OR "contagious duration"~5 OR "contagiousness duration" OR "infectious duration"~5 OR "infectiousness duration"~5 OR "viral duration"~3 OR "CT value" OR "CT values" OR "cycle threshold value" OR "cycle threshold values" OR "spread duration"~5 OR "infectivity duration"~5 OR "infective period"~5 OR "contagious period"~5 OR "infectious period"~5 OR "CT value*" OR "cycle threshold value*" OR "spread period"~5 OR "infectivity period"~5 OR "transmissability period"~5 OR "transmissability duration"~5 OR "transmission period"~5 OR "transmission duration"~5 OR "communicable period"~3 OR "contagion period" OR "communicability period"~3 OR "infectiousness period"~3 OR "contagiousness period"~3 OR "communicability period"~3 OR "shed duration"~5 OR "shedding duration"~5 OR "CT value" OR "cycle threshold values" OR "CT values" OR "cycle threshold values" OR "viral culture" OR "viral cultures" OR "negative culture" OR "viral kinetics"~5 OR "viral time"~5) |
| 2 | ("infective length"~5 OR "contagious length"~5 OR "infectious length"~5 OR "spread length"~5 OR "infectivity length"~5 OR "infective time"~5 OR "contagious time"~5 OR "infectious time"~5 OR "spread time"~5 OR "infectivity time"~5 OR "transmissability length"~5 OR "transmissability time"~5 OR "transmission length"~5 OR "transmission time"~5 OR "shed length"~5 OR "shedding length"~5 OR "shed time"~5 OR "shedding time"~5 OR "communicability length"~5 OR "communicability time"~5) |
| 3 | ti:("infective duration"~5 OR "contagious duration"~5 OR "infectious duration"~5 OR "CT value*" OR "cycle threshold value*" OR "spread duration"~5 OR "infectivity duration"~5 OR "infective period"~5 OR "contagious period"~5 OR "infectious period"~5 OR "spread period"~5 OR "infectivity period"~5 OR "transmissability period"~5 OR "transmissability duration"~5 OR "transmission period"~5 OR "transmission duration"~5 OR "shed duration"~5 OR "shedding duration"~5 OR "CT value" OR "cycle threshold values" OR "CT values" OR "cycle threshold values" OR "infective length"~5 OR "contagious length"~5 OR "infectious length"~5 OR "spread length"~5 OR "infectivity length"~5 OR "infective time"~5 OR "contagious time"~5 OR "infectious time"~5 OR "spread time"~5 OR "infectivity time"~5 OR "transmissability length"~5 OR "transmissability time"~5 OR "transmission length"~5 OR "transmission time"~5 OR "shed length"~5 OR "shedding length"~5 OR "shed time"~5 OR "shedding time"~5 OR "communicability length"~5 OR "communicability time"~5) |
| 4 | #1 OR #2 OR #3 |
| 5 | #4 AND entry_date:([20220101 TO 20220728]) |
| 6 | ((*isolat* OR quarantin*) AND ("antigen test"~3 OR "antigen tests"~3 OR "antigen testing"~3 OR "rapid test"~5 OR "rapid tests"~5 OR "rapid testing"~3 OR "lateral test"~5 OR "lateral tests"~5 OR "lateral testing"~5 OR "lateral flow antigen"~5) ) AND entry_date:([20211123 TO 20220728]) |
| 7 | #5 OR #6 |

# Appendix 2: Methods of the complex microsimulation model

Quilty and colleagues developed a stochastic, individual-based model of different isolation and testing strategies for reducing onward transmission from patients with COVID-19.^1^ We adopted this model and revised it to suit our study purposes. We used data specific to the Omicron variant when available to parameterize the model. Key model parameters are presented in the table below. Using a baseline Ct level of 40, incubation period of 3.42 days,^2^ a peak Ct value of 22.3 for symptomatic individuals,^3^ viral shedding time for symptomatic infections of 19.7 days (95%CI 17.2 to 22.7) and asymptomatic infections of 10.9 days (95% CI 8.3 to 14.3 days),^4^ and day 5, day 6, and day 10 viral culture positivity or rapid antigen test positivity data from our rapid systematic review, we simulated a more accurate viral load trajectory of Ct values over the course of infection for each individual. We assumed that if the Ct value is less than 30, the individual is infectious.^5^

We then applied different isolation strategies and simulated a sample of 10000 individuals. We sampled the proportion of asymptomatic index cases from a beta distribution with a median of 31% (95%CI 24% to 38%).^6^ To estimate secondary cases, we used the basic reproduction number of 2.66 (95% CI 2.41 to 2.94) from a meta-analysis.^7^ We used the 95% CI of the basic reproduction number and uncertainty intervals of hospitalisation rate and fatality rate to calculate 95% uncertainty intervals (UIs). To estimate onward transmission leading to hospitalisation and death, we sampled hospitalisation rate from a beta distribution with a median of 5.54% (95%CI 3.30% to 6.00%) and sampled mortality from a beta distribution with a median of 1.33% (95%CI 0% to 2.00%).^8^

For the isolation of five days and ten days, we estimated hospitalisation and death for secondary cases using both the rapid antigen test data and viral culture data for the model. For the removal of isolation based on a negative antigen test, to estimate hospitalisation and death for secondary cases, we compared the difference between the trajectory of Ct values based on rapid antigen test data and Ct values simulated by viral culture data.

**Table. Key parameters used for the complex microsimulation model**

| **Parameter** | **Value** | **Source** |
| --- | --- | --- |
| Viral culture positivity on day 5 | beta distribution, 37.92%, 95%CI 2.77% to 82.24% | our rapid review |
| Viral culture positivity on day 6 | beta distribution, 28.21%, 95%CI 6.01% to 57.47% | our rapid review |
| Viral culture positivity on day 10 | beta distribution, 0%, 95%CI 0% to 15.07% | our rapid review |
| Rapid antigen test positivity on day 5 | beta distribution, 52.27%, 95%CI 39.11% to 65.27% | our rapid review |
| Rapid antigen test positivity on day 6 | beta distribution, 47.45%, 95%CI 28.2% to 67.09% | our rapid review |
| Rapid antigen test positivity on day 10 | beta distribution, 21.46%, 95%CI 0% to 64.14% | our rapid review |
| Incubation period | log-normal (log-mean 1.12, log-SD 0.41), mean 3.42 days, 95% CI 2.88 to 3.96 days | Wu 2022^2^ |
| Peak Ct value for symptomatic individuals | normally distributed, mean 22.3, SD 4.2 | Kissler 2021^3^ |
| Ct value threshold for an individual that is infectious | Ct value < 30 | Singanayagam 2020^5^ |
| Duration of viral shedding | symptomatic infections: mean 19.7 days, 95%CI 17.2 to 22.7 days; asymptomatic infections: mean 10.9 days, 95% CI 8.3 to 14.3 days | Yan 2021^4^ |
| Asymptomatic fraction of index cases | beta distribution, 31%, 95% CI 24% to 38% | Buitrago-Garcia 2020^6^ |
| Basic reproduction number | 2.66, 95% CI 2.41 to 2.94 | Dhungel 2022^7^ |
| Hospitalisation rate | beta distribution, 5.44%, 95%CI 3.30% to 6.00% | Pitre 2022^8^ |
| Mortality | beta distribution, 1.33%, 95%CI 0 to 2.00% | Pitre 2022^8^ |

References

1. Quilty BJ, Clifford S, Hellewell J, et al. Quarantine and testing strategies in contact tracing for SARS-CoV-2: a modelling study. *Lancet Public Health* 2021; **6**(3): e175-e83.

2. Wu Y, Kang L, Guo Z, Liu J, Liu M, Liang W. Incubation Period of COVID-19 Caused by Unique SARS-CoV-2 Strains: A Systematic Review and Meta-analysis. *JAMA Netw Open* 2022; **5**(8): e2228008.

3. Kissler SM, Fauver JR, Mack C, et al. Viral dynamics of acute SARS-CoV-2 infection and applications to diagnostic and public health strategies. *PLoS Biol* 2021; **19**(7): e3001333.

4. Yan D, Zhang X, Chen C, et al. Characteristics of Viral Shedding Time in SARS-CoV-2 Infections: A Systematic Review and Meta-Analysis. *Front Public Health* 2021; **9**: 652842.

5. Singanayagam A, Patel M, Charlett A, et al. Duration of infectiousness and correlation with RT-PCR cycle threshold values in cases of COVID-19, England, January to May 2020. *Euro Surveill* 2020; **25**(32).

6. Buitrago-Garcia D, Egli-Gany D, Counotte MJ, et al. Occurrence and transmission potential of asymptomatic and presymptomatic SARS-CoV-2 infections: A living systematic review and meta-analysis. *PLoS Med* 2020; **17**(9): e1003346.

7. Dhungel B, Rahman MS, Rahman MM, et al. Reliability of Early Estimates of the Basic Reproduction Number of COVID-19: A Systematic Review and Meta-Analysis. *Int J Environ Res Public Health* 2022; **19**(18).

8. Pitre T, Van Alstine R, Chick G, et al. Antiviral drug treatment for nonsevere COVID-19: a systematic review and network meta-analysis. *CMAJ* 2022; **194**(28): E969-E80.

# Appendix 3. Risk of bias for clinical studies

| **Study** | **Study participants** | **Study attrition** | **Prognostic factor measurement** | **Outcome measurement** | **Statistical analysis and reporting** | **Overall** |
| --- | --- | --- | --- | --- | --- | --- |
| Alshukairi 2022 | Low | Low | Moderate | Low | Low | Low |
| Bouton 2023 | Low | Low | Moderate | Low | Low | Low |
| Cosimi 2022 | Low | Low | Low | Low | Moderate | Low |
| Côté 2022 | Low | Low | Moderate | Low | Moderate | Moderate |
| Earnest 2022 | Low | Low | Low | Low | Low | Low |
| Jang 2022 | Low | Low | Moderate | Low | Moderate | Moderate |
| Jung 2023 | Low | Low | Low | Low | Low | Low |
| Landon 2022 | Low | Low | Moderate | Low | Moderate | Moderate |
| Lefferts 2022 | Low | Low | Low | Low | Moderate | Low |
| Mack 2022 | Low | Low | Moderate | Low | Moderate | Moderate |
| Nelson 2022 | Low | Low | Low | Low | Low | Low |
| Sikka 2022 | Low | Low | Moderate | Low | Low | Low |
| Stingone 2022 | Low | Low | Low | Low | Low | Low |
| Tsao 2022 | Low | Low | Low | Low | Low | Low |
| Wagester 2022 | Moderate | Low | Low | Low | Low | Low |

# Appendix 4. Pooled percentage of rapid antigen test positivity for overall patients

## 4.1. Pooled percentage of rapid antigen test positivity from day 5 to day 8 for overall patients


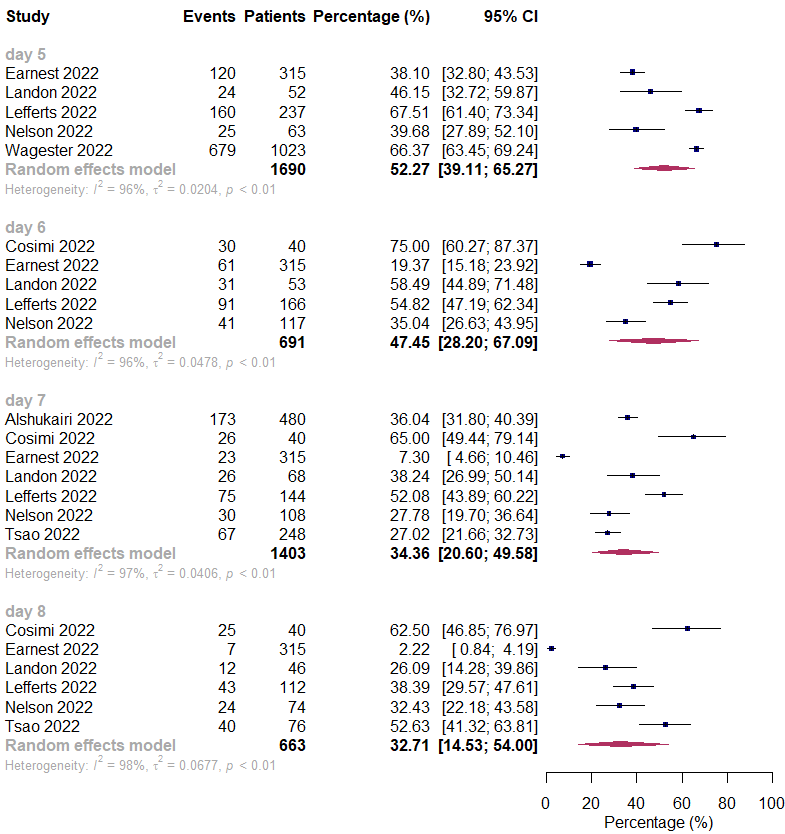


## 4.2. Pooled percentage of rapid antigen test positivity from day 9 to day 14 for overall patients


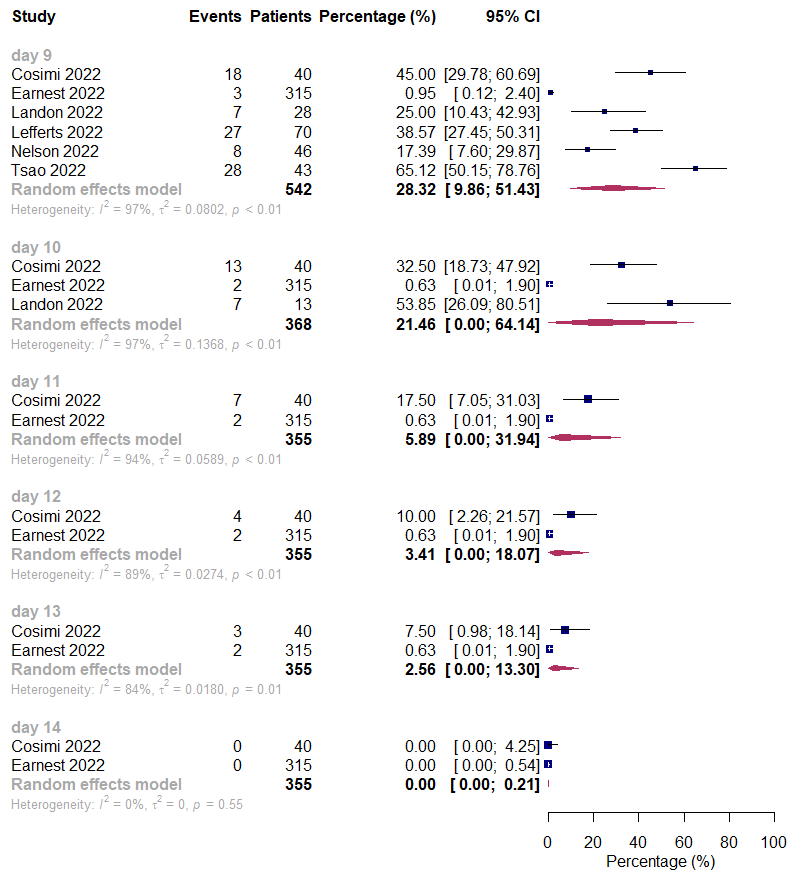


# Appendix 5. GRADE assessment for rapid antigen test positivity and viral culture positivity at days 5, 6 and 10

| **Outcomes** | **№ of studies** | **Effect** | | | **Certainty of the evidence** |
| --- | --- | --- | --- | --- | --- |
|  |  | **№ of events** | **№ of individuals** | **Percentage**  **(95% CI)** |  |
| Day 5 rapid antigen test positivity | 5 | 1008 | 1690 | pooled percentage  52.27 per 100 (39.11 to 65.27) | ⨁⨁⨁◯  Moderate† |
| Day 6 rapid antigen test positivity | 5 | 254 | 691 | pooled percentage  47.45 per 100 (28.20 to 67.09) | ⨁⨁⨁◯  Moderate§ |
| Day 10 rapid antigen test positivity | 3 | 22 | 368 | pooled percentage  21.46 per 100 (0 to 64.14) | ⨁⨁⨁◯  Moderate§ |
| Day 5 viral culture positivity | 2 | 13 | 43 | pooled percentage  37.92 per 100 (2.77 to 82.24) | ⨁⨁◯◯  Low§* |
| Day 6 viral culture positivity | 4 | 28 | 152 | pooled percentage  28.21 per 100 (6.01 to 57.47) | ⨁⨁◯◯  Low§* |
| Day 10 viral culture positivity | 1 | 0 | 11 | pooled percentage  0 per 100 (0 to 15.07) | ⨁⨁◯◯  Low*‡ |

†Rated down one level for inconsistency due to the high heterogeneity.

§Rated down one level for a combination of inconsistency and imprecision.

*Rated down one level for small sample size.

‡Rated down one level for imprecision due to the wide 95% confidence interval.

# Appendix 6. Within-study subgroup analysis of day 8 and day 9 rapid antigen test positivity by symptom status

## 6.1. Within-study subgroup analysis of day 8 rapid antigen test positivity by symptom status


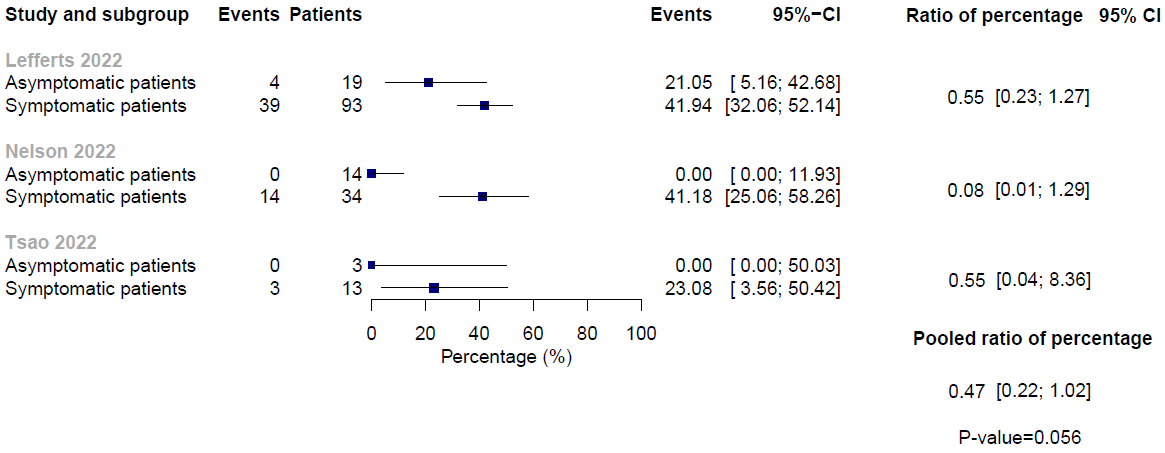


## 6.2. Within-study subgroup analysis of day 9 rapid antigen test positivity by symptom status


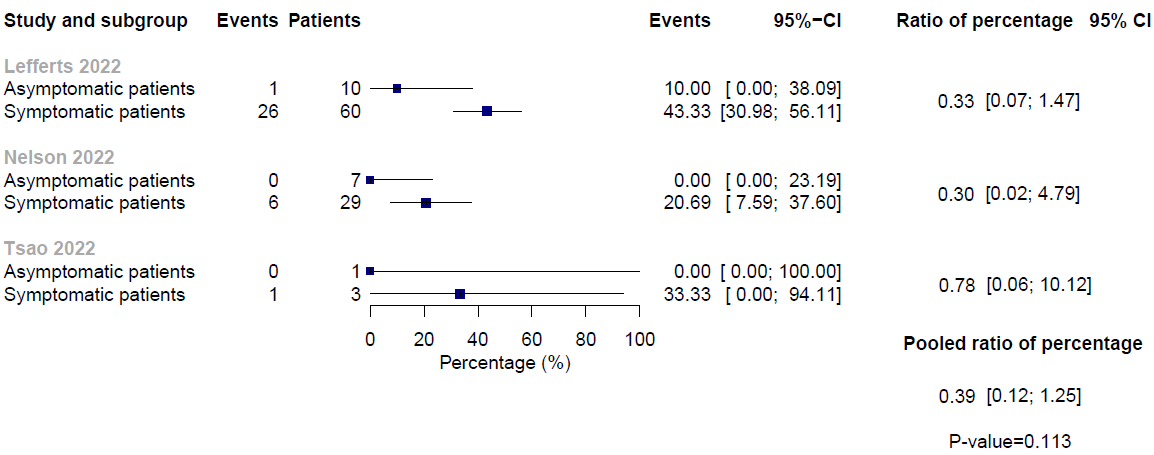


# Appendix 7. Pooled percentage of viral culture positivity from day 5 to day 14


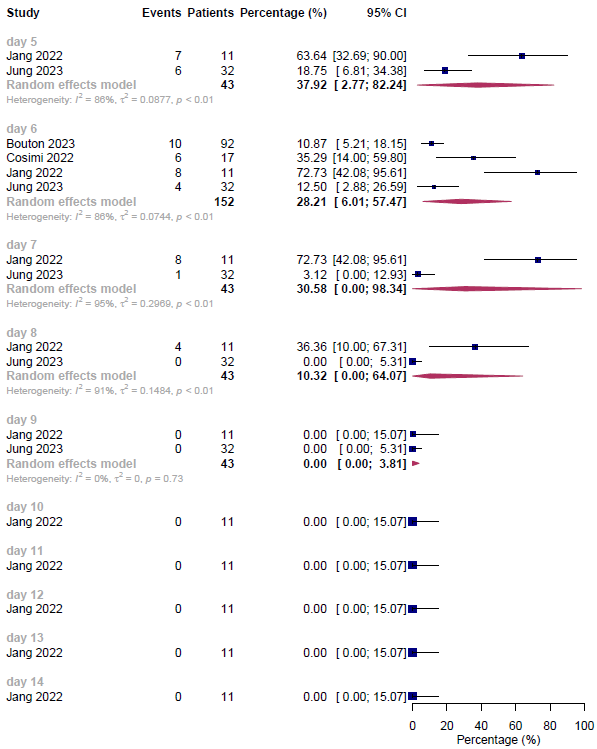


# Appendix 8. Subgroup analysis of day 6 viral culture positivity by symptom status


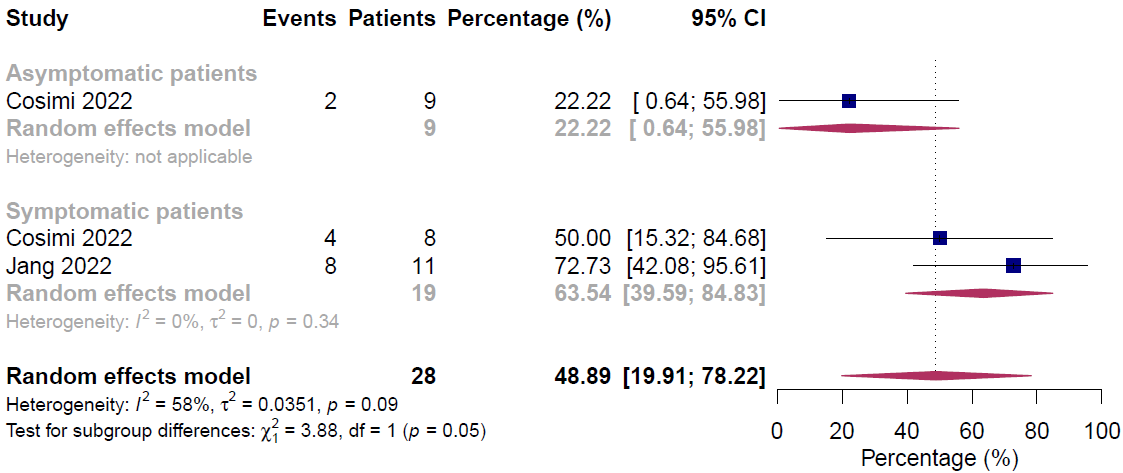


# Appendix 9. Credibility assessment of subgroup effect for day 6 viral culture positivity by symptom status

| **Credibility assessment** | | | |
| --- | --- | --- | --- |
| **1: Is the analysis of effect modification based on comparison within rather than between trials?** | | | |
| [ ] Completely between | [ **X** ] Mostly between or unclear | [ ] Mostly within | [ ] Completely within |
| *Subgroup analysis or meta-regression comparing overall effects of each individual trial. This is typical for aggregate data meta-analysis.* | *Subgroup analysis or meta-regression with most information coming from overall effects, but some trials providing within-trial subgroup information* | *Most trials providing within-trial subgroup information; or individual participant data analysis that combines within and between trial information* | *All trials providing within-trial subgroup information or individual participant data; and the analysis separates within from between trial information, e.g., meta-analysis of interactions* |
| Comment: 1 study provided within study information. | | | |
| **2: For within-trial comparisons, is the effect modification similar from trial to trial?** [ **X** ] Not applicable: no or one within-RCT comparison | | | |
| [ ] Definitely not similar | [ ] Probably not similar or unclear | [ ] Mostly similar | [ ] Definitely similar |
| *Effect modification reported for two or more trials and clearly different directions* | *Effect modification not reported for individual trials or too imprecise to tell* | *Effect modification reported for two or more trials, mostly similar in direction, but considerable differences in magnitude* | *Effect modification reported for two or more trials, similar in direction, only some differences in magnitude* |
| Comment: 1 study provided within study information. | | | |
| **3: For between-trial comparisons, is the number of trials large?** [ ] Not applicable: no between RCT comparison | | | |
| [ **X** ] Very small | [ ] Rather small or unclear | [ ] Rather large | [ ] Large |
| *1 or 2 or in smallest subgroup; 5 or less in continuous meta-regression* | *3-4 in smallest subgroup; 6-10 in continuous meta-regression* | *5-9 in smallest subgroup; 11 to 15 in continuous meta-regression* | *10 or more in smallest subgroup; more than 15 in continuous meta-regression* |
| Comment: 1 study in smallest subgroup. | | | |
| **4: Was the direction of effect modification correctly hypothesized a priori?** | | | |
| [ ] Definitely no | [ ] Probably no or unclear | [ ] Probably yes | [ **X** ] Definitely yes |
| *Clearly post-hoc or results inconsistent with hypothesized direction or biologically very implausible* | *Vague hypothesis or hypothesized direction unclear* | *No prior protocol available but unequivocal statement of a priori hypothesis with correct direction of effect modification* | *Prior protocol available and includes correct specification of direction of effect modification, e.g., based on a biologic rationale* |
| Comment: Positivity rate is lower in asymptomatic patients. | | | |
| **5: Does a test for interaction suggest that chance is an unlikely explanation of the apparent effect modification?** (consider irrespective of number of effect modifiers) | | | |
| [ ] Chance a very likely explanation | [ **X** ] Chance a likely explanation or unclear | [ ] Chance may not explain | [ ] Chance an unlikely explanation |
| *Interaction or meta-regression p-value >0.05* | *Interaction or meta-regression p-value ≤0.05 and >0.01, or no test of interaction reported and not computable* | *Interaction or meta-regression p-value ≤0.01 and >0.005* | *Interaction or meta-regression p-value ≤0.005* |
| Comment: P=0.05 | | | |
| **6: Did the authors test only a small number of effect modifiers or consider the number in their statistical analysis?** | | | |
| [ ] Definitely no | [ ] Probably no or unclear | [ ] Probably yes | [ **X** ] Definitely yes |
| *Explicitly exploratory analysis or large number of effect modifiers tested (e.g., greater than 10) and multiplicity not considered in analysis* | *No mention of number or 4-10 effect modifiers tested and number not considered in analysis* | *No protocol available but unequivocal statement of 3 or fewer effect modifiers tested* | *Protocol available and 3 or fewer effect modifiers tested or number considered in analysis* |
| Comment: Tested 2 effect modifiers. | | | |
| **7: Did the authors use a random effects model?** [ ] Not applicable | | | |
| [ ] Definitely no | [ ] Probably no or unclear | [ ] Probably yes | [ **X** ] Definitely yes |
| *Fixed (or common) effect or fixed effects model explicitly stated* | *Probably fixed effect(s) model* | *Probably random (or mixed) effects* | *Random (or mixed) effects explicitly stated* |
| Comment: Random effects model. | | | |
| **8: If the effect modifier is a continuous variable, were arbitrary cut points avoided?** [ **X** ] not applicable: not continuous | | | |
| [ ] Definitely no | [ ] Probably no or unclear | [ ] Probably yes | [ ] Definitely yes |
| *Analysis based on exploratory cut point(s), e.g., picking cut point associated with highest interaction p-value* | *Analysis based on cut point(s) of unclear origin* | *Analysis based on pre-specified cut point(s), e.g., suggested by prior RCT* | *Analysis based on the full continuum, e.g., assuming a linear or logarithmic relationship* |
| Comment: | | | |
| **9 Optional: Are there any additional considerations that may increase or decrease credibility?** (manual section 3.9) [ **X** ] not applicable | | | |
|  | [ ] Yes, probably decrease  Biologically implausible  Expect similar severe critical  Opposite effects unlikely | [ ] Yes, probably increase | |
| Comment: The cut point for categorization appears to be data driven  The number of events driving the p-value is extremely small  Biology seems very dubious   \| **10: How would you rate the overall credibility of the proposed effect modification?**  The overall rating should be driven by the items that decrease credibility. The following provides a sensible strategy:   - All responses definitely or probably decrease credibility or unclear 🡪 very low - Two or more responses definitely decrease credibility 🡪 maximum usually low even if all other responses satisfy credibility criteria - One response definitely decreases credibility 🡪 maximum usually moderate even if all other responses satisfy credibility criteria - Two responses probably decrease credibility 🡪 maximum usually moderate even if all other responses satisfy credibility criteria - No response options definitely or probably decrease credibility 🡪 high very likely   Place a mark on the continuous line (or type “x” in editable version) \| \| \| \| \|  \| \| --- \| --- \| --- \| --- \| --- \| --- \| \|  \|  \| \| \| \|  \| \|  \| **X** \| \| \| \|  \| \|  \|  \| \|  \|  \| \| \| \|  \| \|  \|  \| \| \| \|  \| \|  \| **Very low credibility** \| **Low credibility** \| **Moderate credibility** \| **High credibility** \|  \| \|  \|  \|  \|  \|  \|  \| \|  \| Very likely no effect modification  Use overall effect for each subgroup \| Likely no effect modification  Use overall effect for each subgroup but note remaining uncertainty \| Likely effect modification  Use separate effects for each subgroup but note remaining uncertainty \| Very likely effect modification  Use separate effects for each subgroup \|  \| \| Comment: Between-trial comparison, the number of studies is small in smallest subgroup, and chance remains a likely explanation for the finding. \| \| \| \| \| \| | | | |

# Appendix 10. Meta-regression analysis by proportion of fully vaccinated patients

## 10.1. Meta-regression analysis for day 5 rapid antigen test positivity by proportion of fully vaccinated patients


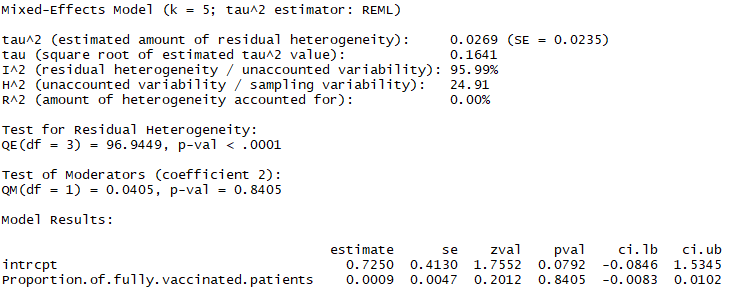


## 10.2. Meta-regression analysis for day 6 rapid antigen test positivity by proportion of fully vaccinated patients


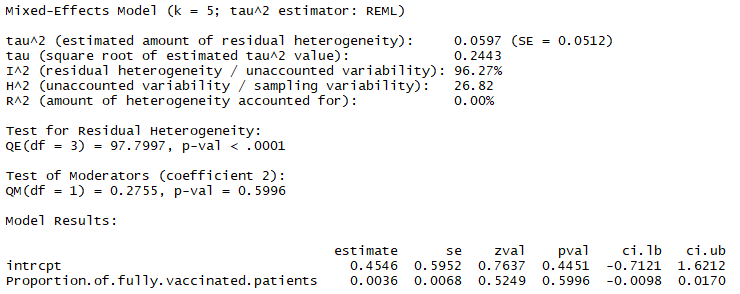


## 10.3. Meta-regression analysis for day 7 rapid antigen test positivity by proportion of fully vaccinated patients


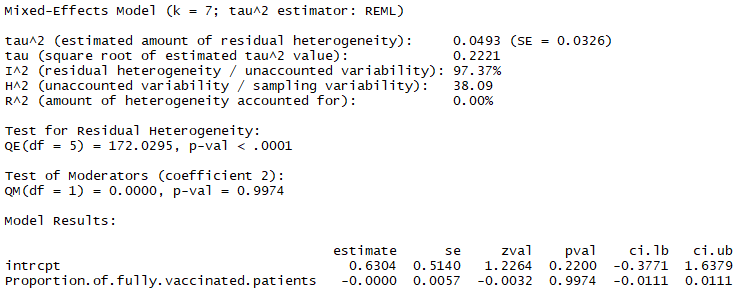


## 10.4. Meta-regression analysis for day 8 rapid antigen test positivity by proportion of fully vaccinated patients


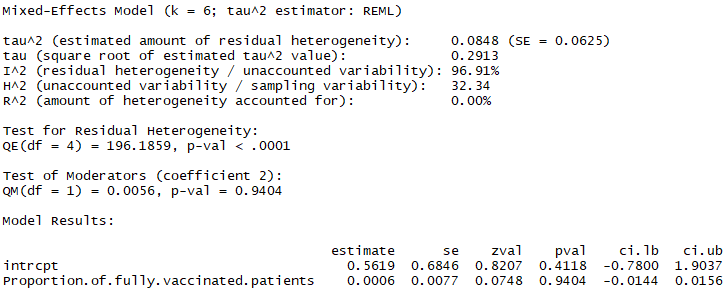


## 10.5. Meta-regression analysis for day 9 rapid antigen test positivity by proportion of fully vaccinated patients


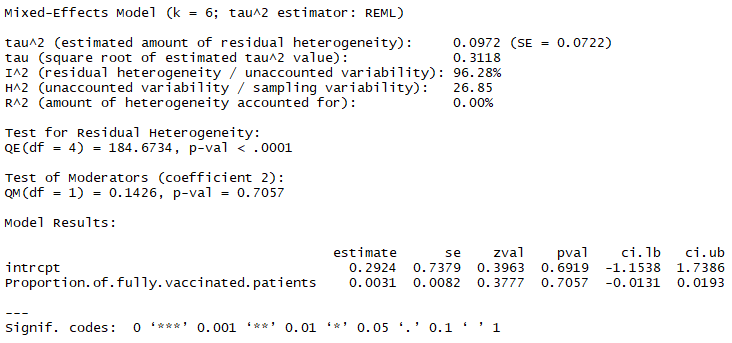


## 10.6. Meta-regression analysis for day 10 rapid antigen test positivity by proportion of fully vaccinated patients


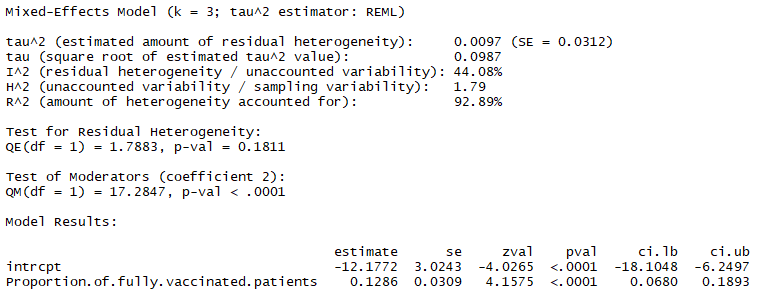


## 10.7. Meta-regression analysis for day 6 viral culture positivity by proportion of fully vaccinated patients


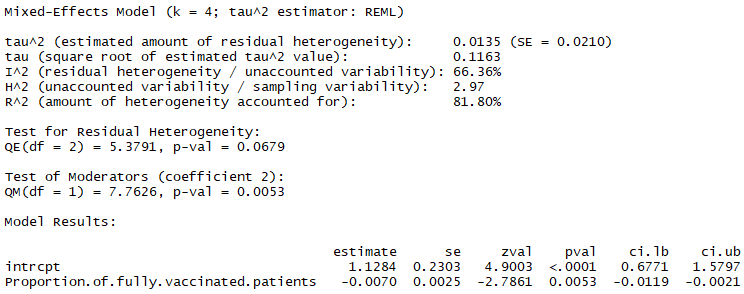


# Appendix 11. Credibility assessment of subgroup effects for rapid antigen test positivity and viral culture positivity by proportion of fully vaccinated patients

## 11.1. Credibility assessment of subgroup analysis for day 10 rapid antigen test positivity by proportion of fully vaccinated patients

| **Credibility assessment** | | | |
| --- | --- | --- | --- |
| **1: Is the analysis of effect modification based on comparison within rather than between trials?** | | | |
| [ **X** ] Completely between | [ ] Mostly between or unclear | [ ] Mostly within | [ ] Completely within |
| *Subgroup analysis or meta-regression comparing overall effects of each individual trial. This is typical for aggregate data meta-analysis.* | *Subgroup analysis or meta-regression with most information coming from overall effects, but some trials providing within-trial subgroup information* | *Most trials providing within-trial subgroup information; or individual participant data analysis that combines within and between trial information* | *All trials providing within-trial subgroup information or individual participant data; and the analysis separates within from between trial information, e.g., meta-analysis of interactions* |
| Comment: No within study information. | | | |
| **2: For within-trial comparisons, is the effect modification similar from trial to trial?** [ **X** ] Not applicable: no or one within-RCT comparison | | | |
| [ ] Definitely not similar | [ ] Probably not similar or unclear | [ ] Mostly similar | [ ] Definitely similar |
| *Effect modification reported for two or more trials and clearly different directions* | *Effect modification not reported for individual trials or too imprecise to tell* | *Effect modification reported for two or more trials, mostly similar in direction, but considerable differences in magnitude* | *Effect modification reported for two or more trials, similar in direction, only some differences in magnitude* |
| Comment: No within study information. | | | |
| **3: For between-trial comparisons, is the number of trials large?** [ ] Not applicable: no between RCT comparison | | | |
| [ **X** ] Very small | [ ] Rather small or unclear | [ ] Rather large | [ ] Large |
| *1 or 2 or in smallest subgroup; 5 or less in continuous meta-regression* | *3-4 in smallest subgroup; 6-10 in continuous meta-regression* | *5-9 in smallest subgroup; 11 to 15 in continuous meta-regression* | *10 or more in smallest subgroup; more than 15 in continuous meta-regression* |
| Comment: 3 studies. | | | |
| **4: Was the direction of effect modification correctly hypothesized a priori?** | | | |
| [ ] Definitely no | [ ] Probably no or unclear | [ ] Probably yes | [ **X** ] Definitely yes |
| *Clearly post-hoc or results inconsistent with hypothesized direction or biologically very implausible* | *Vague hypothesis or hypothesized direction unclear* | *No prior protocol available but unequivocal statement of a priori hypothesis with correct direction of effect modification* | *Prior protocol available and includes correct specification of direction of effect modification, e.g., based on a biologic rationale* |
| Comment: A lower positive rate in studies with a higher proportion fully vaccinated patient. | | | |
| **5: Does a test for interaction suggest that chance is an unlikely explanation of the apparent effect modification?** (consider irrespective of number of effect modifiers) | | | |
| [ ] Chance a very likely explanation | [ ] Chance a likely explanation or unclear | [ ] Chance may not explain | [ **X** ] Chance an unlikely explanation |
| *Interaction or meta-regression p-value >0.05* | *Interaction or meta-regression p-value ≤0.05 and >0.01, or no test of interaction reported and not computable* | *Interaction or meta-regression p-value ≤0.01 and >0.005* | *Interaction or meta-regression p-value ≤0.005* |
| Comment: P<0.001 | | | |
| **6: Did the authors test only a small number of effect modifiers or consider the number in their statistical analysis?** | | | |
| [ ] Definitely no | [ ] Probably no or unclear | [ ] Probably yes | [ **X** ] Definitely yes |
| *Explicitly exploratory analysis or large number of effect modifiers tested (e.g., greater than 10) and multiplicity not considered in analysis* | *No mention of number or 4-10 effect modifiers tested and number not considered in analysis* | *No protocol available but unequivocal statement of 3 or fewer effect modifiers tested* | *Protocol available and 3 or fewer effect modifiers tested or number considered in analysis* |
| Comment: Tested 2 effect modifiers. | | | |
| **7: Did the authors use a random effects model?** [ ] Not applicable | | | |
| [ ] Definitely no | [ ] Probably no or unclear | [ ] Probably yes | [ **X** ] Definitely yes |
| *Fixed (or common) effect or fixed effects model explicitly stated* | *Probably fixed effect(s) model* | *Probably random (or mixed) effects* | *Random (or mixed) effects explicitly stated* |
| Comment: Random effects model. | | | |
| **8: If the effect modifier is a continuous variable, were arbitrary cut points avoided?** [ ] not applicable: not continuous | | | |
| [ ] Definitely no | [ ] Probably no or unclear | [ ] Probably yes | [ **X** ] Definitely yes |
| *Analysis based on exploratory cut point(s), e.g., picking cut point associated with highest interaction p-value* | *Analysis based on cut point(s) of unclear origin* | *Analysis based on pre-specified cut point(s), e.g., suggested by prior RCT* | *Analysis based on the full continuum, e.g., assuming a linear or logarithmic relationship* |
| Comment: | | | |
| **9 Optional: Are there any additional considerations that may increase or decrease credibility?** (manual section 3.9) [ **X** ] not applicable | | | |
|  | [ ] Yes, probably decrease  Biologically implausible  Expect similar severe critical  Opposite effects unlikely | [ ] Yes, probably increase | |
| Comment: The cut point for categorization appears to be data driven  The number of events driving the p-value is extremely small  Biology seems very dubious   \| **10: How would you rate the overall credibility of the proposed effect modification?**  The overall rating should be driven by the items that decrease credibility. The following provides a sensible strategy:   - All responses definitely or probably decrease credibility or unclear 🡪 very low - Two or more responses definitely decrease credibility 🡪 maximum usually low even if all other responses satisfy credibility criteria - One response definitely decreases credibility 🡪 maximum usually moderate even if all other responses satisfy credibility criteria - Two responses probably decrease credibility 🡪 maximum usually moderate even if all other responses satisfy credibility criteria - No response options definitely or probably decrease credibility 🡪 high very likely   Place a mark on the continuous line (or type “x” in editable version) \| \| \| \| \|  \| \| --- \| --- \| --- \| --- \| --- \| --- \| \|  \|  \| \| \| \|  \| \|  \| **X** \| \| \| \|  \| \|  \|  \| \|  \|  \| \| \| \|  \| \|  \|  \| \| \| \|  \| \|  \| **Very low credibility** \| **Low credibility** \| **Moderate credibility** \| **High credibility** \|  \| \|  \|  \|  \|  \|  \|  \| \|  \| Very likely no effect modification  Use overall effect for each subgroup \| Likely no effect modification  Use overall effect for each subgroup but note remaining uncertainty \| Likely effect modification  Use separate effects for each subgroup but note remaining uncertainty \| Very likely effect modification  Use separate effects for each subgroup \|  \| \| Comment: Between-trial comparison, the number of studies is small. \| \| \| \| \| \| | | | |

## 11.2. Credibility assessment of subgroup analysis for day 6 viral culture positivity by proportion of fully vaccinated patients

| **Credibility assessment** | | | |
| --- | --- | --- | --- |
| **1: Is the analysis of effect modification based on comparison within rather than between trials?** | | | |
| [ **X** ] Completely between | [ ] Mostly between or unclear | [ ] Mostly within | [ ] Completely within |
| *Subgroup analysis or meta-regression comparing overall effects of each individual trial. This is typical for aggregate data meta-analysis.* | *Subgroup analysis or meta-regression with most information coming from overall effects, but some trials providing within-trial subgroup information* | *Most trials providing within-trial subgroup information; or individual participant data analysis that combines within and between trial information* | *All trials providing within-trial subgroup information or individual participant data; and the analysis separates within from between trial information, e.g., meta-analysis of interactions* |
| Comment: No within study information. | | | |
| **2: For within-trial comparisons, is the effect modification similar from trial to trial?** [ **X** ] Not applicable: no or one within-RCT comparison | | | |
| [ ] Definitely not similar | [ ] Probably not similar or unclear | [ ] Mostly similar | [ ] Definitely similar |
| *Effect modification reported for two or more trials and clearly different directions* | *Effect modification not reported for individual trials or too imprecise to tell* | *Effect modification reported for two or more trials, mostly similar in direction, but considerable differences in magnitude* | *Effect modification reported for two or more trials, similar in direction, only some differences in magnitude* |
| Comment: No within study information. | | | |
| **3: For between-trial comparisons, is the number of trials large?** [ ] Not applicable: no between RCT comparison | | | |
| [ **X** ] Very small | [ ] Rather small or unclear | [ ] Rather large | [ ] Large |
| *1 or 2 or in smallest subgroup; 5 or less in continuous meta-regression* | *3-4 in smallest subgroup; 6-10 in continuous meta-regression* | *5-9 in smallest subgroup; 11 to 15 in continuous meta-regression* | *10 or more in smallest subgroup; more than 15 in continuous meta-regression* |
| Comment: 4 studies. | | | |
| **4: Was the direction of effect modification correctly hypothesized a priori?** | | | |
| [ ] Definitely no | [ ] Probably no or unclear | [ ] Probably yes | [ **X** ] Definitely yes |
| *Clearly post-hoc or results inconsistent with hypothesized direction or biologically very implausible* | *Vague hypothesis or hypothesized direction unclear* | *No prior protocol available but unequivocal statement of a priori hypothesis with correct direction of effect modification* | *Prior protocol available and includes correct specification of direction of effect modification, e.g., based on a biologic rationale* |
| Comment: A lower positive rate in studies with a higher proportion fully vaccinated patient. | | | |
| **5: Does a test for interaction suggest that chance is an unlikely explanation of the apparent effect modification?** (consider irrespective of number of effect modifiers) | | | |
| [ ] Chance a very likely explanation | [ ] Chance a likely explanation or unclear | [ **X** ] Chance may not explain | [ ] Chance an unlikely explanation |
| *Interaction or meta-regression p-value >0.05* | *Interaction or meta-regression p-value ≤0.05 and >0.01, or no test of interaction reported and not computable* | *Interaction or meta-regression p-value ≤0.01 and >0.005* | *Interaction or meta-regression p-value ≤0.005* |
| Comment: P=0.0053 | | | |
| **6: Did the authors test only a small number of effect modifiers or consider the number in their statistical analysis?** | | | |
| [ ] Definitely no | [ ] Probably no or unclear | [ ] Probably yes | [ **X** ] Definitely yes |
| *Explicitly exploratory analysis or large number of effect modifiers tested (e.g., greater than 10) and multiplicity not considered in analysis* | *No mention of number or 4-10 effect modifiers tested and number not considered in analysis* | *No protocol available but unequivocal statement of 3 or fewer effect modifiers tested* | *Protocol available and 3 or fewer effect modifiers tested or number considered in analysis* |
| Comment: Tested 2 effect modifiers. | | | |
| **7: Did the authors use a random effects model?** [ ] Not applicable | | | |
| [ ] Definitely no | [ ] Probably no or unclear | [ ] Probably yes | [ **X** ] Definitely yes |
| *Fixed (or common) effect or fixed effects model explicitly stated* | *Probably fixed effect(s) model* | *Probably random (or mixed) effects* | *Random (or mixed) effects explicitly stated* |
| Comment: Random effects model. | | | |
| **8: If the effect modifier is a continuous variable, were arbitrary cut points avoided?** [ ] not applicable: not continuous | | | |
| [ ] Definitely no | [ ] Probably no or unclear | [ ] Probably yes | [ **X** ] Definitely yes |
| *Analysis based on exploratory cut point(s), e.g., picking cut point associated with highest interaction p-value* | *Analysis based on cut point(s) of unclear origin* | *Analysis based on pre-specified cut point(s), e.g., suggested by prior RCT* | *Analysis based on the full continuum, e.g., assuming a linear or logarithmic relationship* |
| Comment: | | | |
| **9 Optional: Are there any additional considerations that may increase or decrease credibility?** (manual section 3.9) [ **X** ] not applicable | | | |
|  | [ ] Yes, probably decrease  Biologically implausible  Expect similar severe critical  Opposite effects unlikely | [ ] Yes, probably increase | |
| Comment: The cut point for categorization appears to be data driven  The number of events driving the p-value is extremely small  Biology seems very dubious   \| **10: How would you rate the overall credibility of the proposed effect modification?**  The overall rating should be driven by the items that decrease credibility. The following provides a sensible strategy:   - All responses definitely or probably decrease credibility or unclear 🡪 very low - Two or more responses definitely decrease credibility 🡪 maximum usually low even if all other responses satisfy credibility criteria - One response definitely decreases credibility 🡪 maximum usually moderate even if all other responses satisfy credibility criteria - Two responses probably decrease credibility 🡪 maximum usually moderate even if all other responses satisfy credibility criteria - No response options definitely or probably decrease credibility 🡪 high very likely   Place a mark on the continuous line (or type “x” in editable version) \| \| \| \| \|  \| \| --- \| --- \| --- \| --- \| --- \| --- \| \|  \|  \| \| \| \|  \| \|  \| **X** \| \| \| \|  \| \|  \|  \| \|  \|  \| \| \| \|  \| \|  \|  \| \| \| \|  \| \|  \| **Very low credibility** \| **Low credibility** \| **Moderate credibility** \| **High credibility** \|  \| \|  \|  \|  \|  \|  \|  \| \|  \| Very likely no effect modification  Use overall effect for each subgroup \| Likely no effect modification  Use overall effect for each subgroup but note remaining uncertainty \| Likely effect modification  Use separate effects for each subgroup but note remaining uncertainty \| Very likely effect modification  Use separate effects for each subgroup \|  \| \| Comment: Between-trial comparison, the number of studies is small. \| \| \| \| \| \| | | | |

# Appendix 12. Sensitivity analyses only including studies with low risk of bias

## 12.1. Sensitivity analyses for day 5 to day 8 rapid antigen test positivity only including studies with low risk of bias


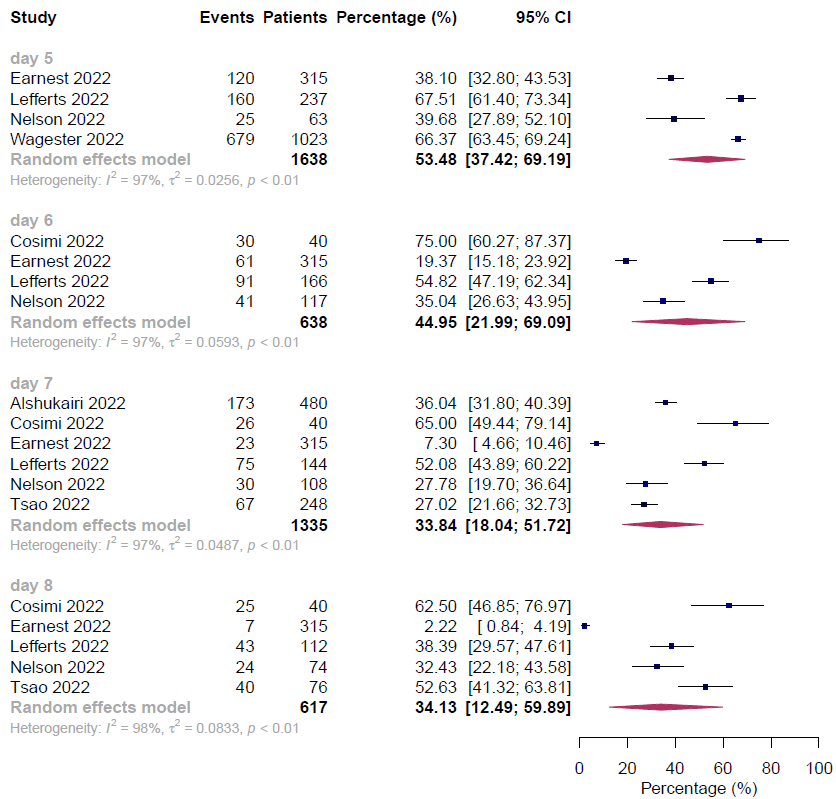


## 12.2. Sensitivity analyses for day 9 to day 14 rapid antigen test positivity only including studies with low risk of bias


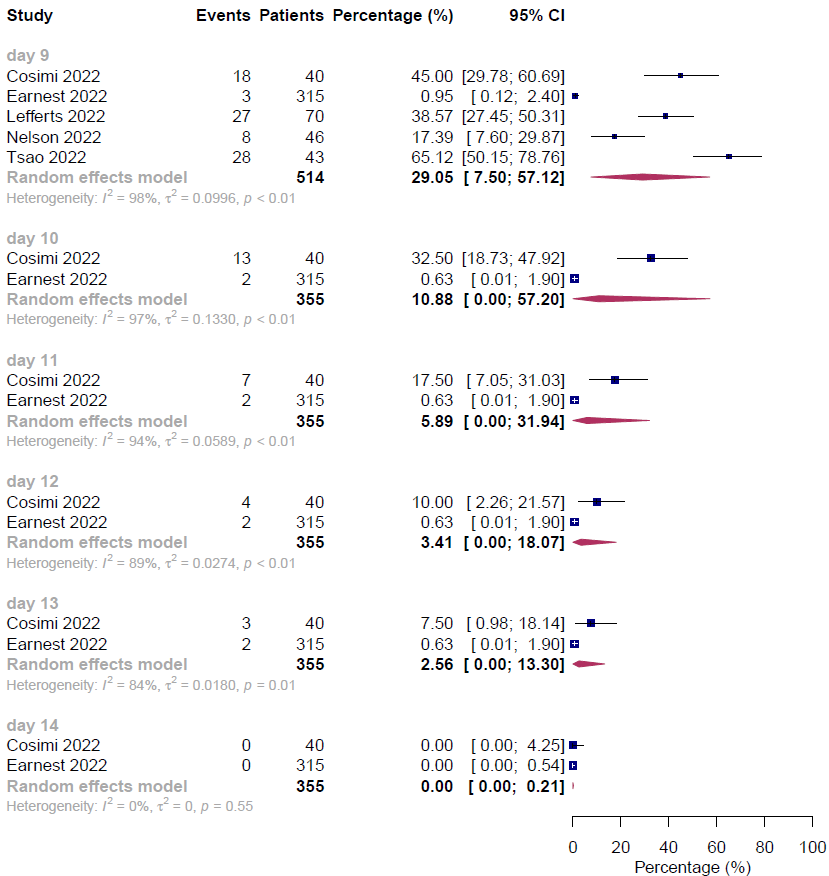


## 12.3. Sensitivity analyses for day 5 to day 14 viral culture positivity only including studies with low risk of bias


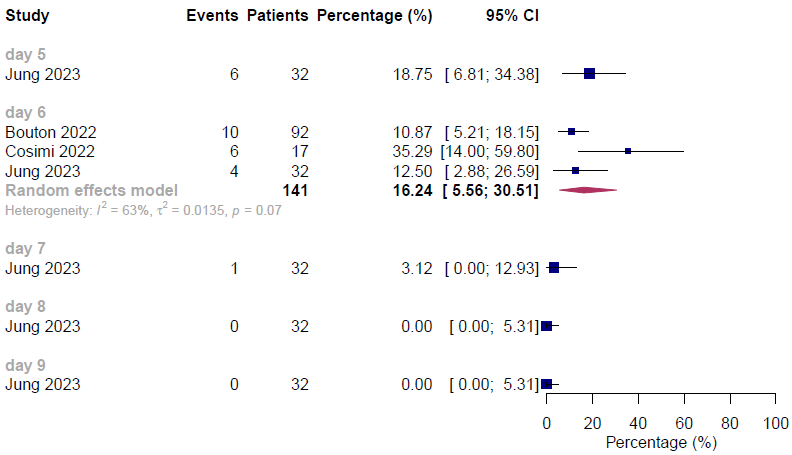


# Appendix 13. Additional results of rapid systematic review

One study^1^, including 10 severe patients with alpha variant, reported that isolation duration exceeded 21 days for seven patients and two of them have positive viral cultures on day 21. In one study^2^ with 32 patients, the percentage of PCR positivity from day 5 to day 9 varied from 84.4% to 3.1%. In one study^3^ with 173 fully vaccinated patients, 45.7% of patients had at least one negative or Ct ≥ 35 RT-PCR test result on or before day 6 and the median time from diagnosis to first negative result was 7 days (IQR 5 to 9 days). In one study^4^ with 37 patients, the percentage of PCR-positive with Ct value < 30 from day 5 to day 10 varied from 51.4% to 8.1% and the average time to PCR clearance was 7.94 days. One study^5^ with 196 SARS-CoV-2 positive homeless people reported that 26.5% of patients had a positive PCR test after 21 days of isolation and the median duration of positivity was 21 days (IQR 14 to 26).

References

1. Côté F, Boivin G, Loungnarath V, et al. Duration of isolation and contagiousness in COVID-19 patients receiving tocilizumab and dexamethasone: a case series. Infect Control Hosp Epidemiol 2022: doi: 10.1017/ice.2022.14.

2. Jung J, Kang SW, Lee S, et al. Risk of transmission of COVID-19 from healthcare workers returning to work after a 5-day isolation, and kinetics of shedding of viable SARS-CoV-2 variant B.1.1.529 (Omicron). J Hosp Infect 2023; 131: 228-33.

3. Mack CD, Wasserman EB, Killerby ME, et al. Results from a Test-to-Release from Isolation Strategy Among Fully Vaccinated National Football League Players and Staff Members with COVID-19 - United States, December 14-19, 2021. MMWR Morb Mortal Wkly Rep 2022; 71(8): 299-305.

4. Sikka R, Wyllie AL, Premsrirut P, Berke EM. COVID Testing in the Workplace: Return to Work Testing in an Occupational Cohort. medRxiv 2022: doi: https://doi.org/10.1101/2022.02.09.22270653.

5. Stingone C, Latini A, Gianserra L, et al. Features of fragile people with SARS-CoV-2 infection in isolation in a COVID-19 hotel in Rome, Italy. European Review for Medical and Pharmacological Sciences 2022; 26(7): 2631-8.

# Appendix 14. GRADE summary of findings for five-day isolation versus ten-day isolation for outcomes estimated using positive viral culture data from relatively simple model for overall patients

| **Outcome** | **Absolute effect estimates** | | **Certainty of the evidence** | **Plain language summary** |
| --- | --- | --- | --- | --- |
|  | Isolation for 5 days | Isolation for 10 days |  |  |
| **We assume that all patients with positive viral culture are infectious and that isolation adherence is 100%** | | | | |
| Onward transmission leading to hospitalisation (28 days) | **151**  per 10000 | **0**  per 10000 | **Very low**  Due to certainty of parameters (low) in the model and indirectness | Whether isolation of 5 days compared with 10 days would increase hospitalisation for secondary cases is very uncertain. |
|  | Difference: **151 more per 10000**  (95% UI 91 more to 224 more) | |  |  |
| Onward transmission leading to death (90 days) | **33**  per 10000 | **0**  per 10000 | **Very low**  Due to certainty of parameters (low) in the model and indirectness | Whether isolation of 5 days compared with 10 days would increase mortality for secondary cases is very uncertain. |
|  | Difference: **33 more per 10000**  (95% UI 9 more to 59 more) | |  |  |
| **We assume that all patients with positive viral culture are infectious and that isolation adherence is 80%** | | | | |
| Onward transmission leading to hospitalisation (28 days) | **200**  per 10000 | **79**  per 10000 | **Very low**  Due to certainty of parameters (low) in the model and indirectness | Whether isolation of 5 days compared with 10 days would increase hospitalisation for secondary cases is very uncertain. |
|  | Difference: **121 more per 10000**  (95% UI 73 more to 179 more) | |  |  |
| Onward transmission leading to death (90 days) | **44**  per 10000 | **17**  per 10000 | **Very low**  Due to certainty of parameters (low) in the model and indirectness | Whether isolation of 5 days compared with 10 days would increase mortality for secondary cases is very uncertain. |
|  | Difference: **27 more per 10000**  (95% UI 7 more to 47 more) | |  |  |
| **We assume that all patients with positive viral culture are infectious and that isolation adherence is 50%** | | | | |
| Onward transmission leading to hospitalisation (28 days) | **274**  per 10000 | **199**  per 10000 | **Very low**  Due to certainty of parameters (low) in the model and indirectness | Whether isolation of 5 days compared with 10 days would increase hospitalisation for secondary cases is very uncertain. |
|  | Difference: **75 more per 10000**  (95% UI 46 more to 112 more) | |  |  |
| Onward transmission leading to death (90 days) | **60**  per 10000 | **44**  per 10000 | **Very low**  Due to certainty of parameters (low) in the model and indirectness | Whether isolation of 5 days compared with 10 days would increase mortality for secondary cases is very uncertain. |
|  | Difference: **16 more per 10000**  (95% UI 5 more to 30 more) | |  |  |

UI, uncertainty interval.

# **Appendix 15**. Sensitivity analysis of five-day isolation versus ten-day isolation for outcomes estimated using rapid antigen test data from relatively simple model considering different isolation adherence rates and false negative rates (GRADE summary of findings)

## **15.1.** Sensitivity analysis of five-day isolation versus ten-day isolation for outcomes estimated using rapid antigen test data from relatively simple model using an isolation adherence of 80% and a false negative rate of 20%

| **Outcome** | **Absolute effect estimates** | | **Certainty of the evidence** | **Plain language summary** |
| --- | --- | --- | --- | --- |
|  | Isolation for 5 days | Isolation for 10 days |  |  |
| **All patients** | | | | |
| Onward transmission leading to hospitalisation (28 days) | **279**  per 10000 | **161**  per 10000 | **Very low**  Due to certainty of parameters (moderate) in the model and indirectness | Whether isolation of 5 days compared with 10 days would increase onward transmission leading to hospitalisation for secondary cases is very uncertain. |
|  | Difference: **118 more per 10000**  (95% UI 71 more to 175 more) | |  |  |
| Onward transmission leading to death (90 days) | **61**  per 10000 | **35**  per 10000 | **Very low**  Due to certainty of parameters (moderate) in the model and indirectness | Whether isolation of 5 days compared with 10 days would increase onward transmission leading to death for secondary cases is very uncertain. |
|  | Difference: **26 more per 10000**  (95% UI 7 more to 46 more) | |  |  |
| **Asymptomatic patients** | | | | |
| Onward transmission leading to hospitalisation (28 days) | **183**  per 10000 | **161**  per 10000 | **Very Low**  Due to certainty of parameters (moderate) in the model and indirectness | Whether isolation for 5 days compared with 10 days would increase onward transmission leading to hospitalisation of secondary cases is very uncertain. |
|  | Difference: **22 more per 10000**  (95% UI 13 more to 32 more) | |  |  |
| Onward transmission leading to death (90 days) | **40**  per 10000 | **35**  per 10000 | **Very Low**  Due to certainty of parameters (moderate) in the model and indirectness | Whether isolation for 5 days compared with 10 days would increase onward transmission leading to death of secondary cases is very uncertain. |
|  | Difference: **5 more per 10000**  (95% UI 1 more to 8 more) | |  |  |
| **Symptomatic patients** | | | | |
| Onward transmission leading to hospitalisation (28 days) | **339**  per 10000 | **161**  per 10000 | **Very Low**  Due to certainty of parameters (moderate) in the model and indirectness | Whether isolation for 5 days compared with 10 days would increase onward transmission leading to hospitalisation of secondary cases is very uncertain. |
|  | Difference: **178 more per 10000**  (95% UI 108 more to 265 more) | |  |  |
| Onward transmission leading to death (90 days) | **75**  per 10000 | **35**  per 10000 | **Very Low**  Due to certainty of parameters (moderate) in the model and indirectness | Whether isolation for 5 days compared with 10 days would increase onward transmission leading to death of secondary cases is very uncertain. |
|  | Difference: **40 more per 10000**  (95% UI 11 to 70 more) | |  |  |

UI, uncertainty interval.

## **15.2.** Sensitivity analysis of five-day isolation versus ten-day isolation for outcomes estimated using rapid antigen test data from relatively simple model using an isolation adherence of 80% and a false negative rate of 33%

| **Outcome** | **Absolute effect estimates** | | **Certainty of the evidence** | **Plain language summary** |
| --- | --- | --- | --- | --- |
|  | Isolation for 5 days | Isolation for 10 days |  |  |
| **All patients** | | | | |
| Onward transmission leading to hospitalisation (28 days) | **301**  per 10000 | **170**  per 10000 | **Very low**  Due to certainty of parameters (moderate) in the model and indirectness | Whether isolation of 5 days compared with 10 days would increase onward transmission leading to hospitalisation for secondary cases is very uncertain. |
|  | Difference: **131 more per 10000**  (95% UI 79 more to 194 more) | |  |  |
| Onward transmission leading to death (90 days) | **66**  per 10000 | **37**  per 10000 | **Very low**  Due to certainty of parameters (moderate) in the model and indirectness | Whether isolation of 5 days compared with 10 days would increase onward transmission leading to death for secondary cases is very uncertain. |
|  | Difference: **29 more per 10000**  (95% UI 8 more to 51 more) | |  |  |
| **Asymptomatic patients** | | | | |
| Onward transmission leading to hospitalisation (28 days) | **194**  per 10000 | **170**  per 10000 | **Very Low**  Due to certainty of parameters (moderate) in the model and indirectness | Whether isolation for 5 days compared with 10 days would increase onward transmission leading to hospitalisation of secondary cases is very uncertain. |
|  | Difference: **24 more per 10000**  (95% UI 15 more to 36 more) | |  |  |
| Onward transmission leading to death (90 days) | **43**  per 10000 | **37**  per 10000 | **Very Low**  Due to certainty of parameters (moderate) in the model and indirectness | Whether isolation for 5 days compared with 10 days would increase onward transmission leading to death of secondary cases is very uncertain. |
|  | Difference: **6 more per 10000**  (95% UI 1 more to 9 more) | |  |  |
| **Symptomatic patients** | | | | |
| Onward transmission leading to hospitalisation (28 days) | **368**  per 10000 | **170**  per 10000 | **Very Low**  Due to certainty of parameters (moderate) in the model and indirectness | Whether isolation for 5 days compared with 10 days would increase onward transmission leading to hospitalisation of secondary cases is very uncertain. |
|  | Difference: **198 more per 10000**  (95% UI 120 more to 294 more) | |  |  |
| Onward transmission leading to death (90 days) | **81**  per 10000 | **37**  per 10000 | **Very Low**  Due to certainty of parameters (moderate) in the model and indirectness | Whether isolation for 5 days compared with 10 days would increase onward transmission leading to death of secondary cases is very uncertain. |
|  | Difference: **44 more per 10000**  (95% UI 12 to 77 more) | |  |  |

UI, uncertainty interval.

## **15.3.** Sensitivity analysis of five-day isolation versus ten-day isolation for outcomes estimated using rapid antigen test data from relatively simple model using an isolation adherence of 50% and a false negative rate of 20%

| **Outcome** | **Absolute effect estimates** | | **Certainty of the evidence** | **Plain language summary** |
| --- | --- | --- | --- | --- |
|  | Isolation for 5 days | Isolation for 10 days |  |  |
| **All patients** | | | | |
| Onward transmission leading to hospitalisation (28 days) | **323**  per 10000 | **250**  per 10000 | **Very low**  Due to certainty of parameters (moderate) in the model and indirectness | Whether isolation of 5 days compared with 10 days would increase onward transmission leading to hospitalisation for secondary cases is very uncertain. |
|  | Difference: **73 more per 10000**  (95% UI 45 more to 109 more) | |  |  |
| Onward transmission leading to death (90 days) | **71**  per 10000 | **55**  per 10000 | **Very low**  Due to certainty of parameters (moderate) in the model and indirectness | Whether isolation of 5 days compared with 10 days would increase onward transmission leading to death for secondary cases is very uncertain. |
|  | Difference: **16 more per 10000**  (95% UI 4 more to 29 more) | |  |  |
| **Asymptomatic patients** | | | | |
| Onward transmission leading to hospitalisation (28 days) | **263**  per 10000 | **250**  per 10000 | **Very Low**  Due to certainty of parameters (moderate) in the model and indirectness | Whether isolation for 5 days compared with 10 days would increase onward transmission leading to hospitalisation of secondary cases is very uncertain. |
|  | Difference: **13 more per 10000**  (95% UI 8 more to 20 more) | |  |  |
| Onward transmission leading to death (90 days) | **58**  per 10000 | **55**  per 10000 | **Very Low**  Due to certainty of parameters (moderate) in the model and indirectness | Whether isolation for 5 days compared with 10 days would increase onward transmission leading to death of secondary cases is very uncertain. |
|  | Difference: **3 more per 10000**  (95% UI 1 more to 5 more) | |  |  |
| **Symptomatic patients** | | | | |
| Onward transmission leading to hospitalisation (28 days) | **361**  per 10000 | **250**  per 10000 | **Very Low**  Due to certainty of parameters (moderate) in the model and indirectness | Whether isolation for 5 days compared with 10 days would increase onward transmission leading to hospitalisation of secondary cases is very uncertain. |
|  | Difference: **111 more per 10000**  (95% UI 68 more to 166 more) | |  |  |
| Onward transmission leading to death (90 days) | **79**  per 10000 | **55**  per 10000 | **Very Low**  Due to certainty of parameters (moderate) in the model and indirectness | Whether isolation for 5 days compared with 10 days would increase onward transmission leading to death of secondary cases is very uncertain. |
|  | Difference: **24 more per 10000**  (95% UI 7 to 44 more) | |  |  |

UI, uncertainty interval.

## **15.4.** Sensitivity analysis of five-day isolation versus ten-day isolation for outcomes estimated using rapid antigen test data from relatively simple model using an isolation adherence of 50% and a false negative rate of 33%

| **Outcome** | **Absolute effect estimates** | | **Certainty of the evidence** | **Plain language summary** |  |
| --- | --- | --- | --- | --- | --- |
|  | Isolation for 5 days | Isolation for 10 days |  |  |  |
| **All patients** | | | | | |
| Onward transmission leading to hospitalisation (28 days) | **337**  per 10000 | **255**  per 10000 | **Very low**  Due to certainty of parameters (moderate) in the model and indirectness | Whether isolation of 5 days compared with 10 days would increase onward transmission leading to hospitalisation for secondary cases is very uncertain. |  |
|  | Difference: **82 more per 10000**  (95% UI 49 more to 121 more) | |  |  |  |
| Onward transmission leading to death (90 days) | **74**  per 10000 | **56**  per 10000 | **Very low**  Due to certainty of parameters (moderate) in the model and indirectness | Whether isolation of 5 days compared with 10 days would increase onward transmission leading to death for secondary cases is very uncertain. |  |
|  | Difference: **18 more per 10000**  (95% UI 5 more to 32 more) | |  |  |  |
| **Asymptomatic patients** | | | | | |
| Onward transmission leading to hospitalisation (28 days) | **270**  per 10000 | **255**  per 10000 | **Very Low**  Due to certainty of parameters (moderate) in the model and indirectness | Whether isolation for 5 days compared with 10 days would increase onward transmission leading to hospitalisation of secondary cases is very uncertain. |  |
|  | Difference: **15 more per 10000**  (95% UI 9 more to 22 more) | |  |  |  |
| Onward transmission leading to death (90 days) | **59**  per 10000 | **56**  per 10000 | **Very Low**  Due to certainty of parameters (moderate) in the model and indirectness | Whether isolation for 5 days compared with 10 days would increase onward transmission leading to death of secondary cases is very uncertain. |  |
|  | Difference: **3 more per 10000**  (95% UI 1 more to 6 more) | |  |  |  |
| **Symptomatic patients** | | | | | |
| Onward transmission leading to hospitalisation (28 days) | **379**  per 10000 | **255**  per 10000 | **Very Low**  Due to certainty of parameters (moderate) in the model and indirectness | Whether isolation for 5 days compared with 10 days would increase onward transmission leading to hospitalisation of secondary cases is very uncertain. |  |
|  | Difference: **124 more per 10000**  (95% UI 75 more to 184 more) | |  |  |  |
| Onward transmission leading to death (90 days) | **83**  per 10000 | **56**  per 10000 | **Very Low**  Due to certainty of parameters (moderate) in the model and indirectness | Whether isolation for 5 days compared with 10 days would increase onward transmission leading to death of secondary cases is very uncertain. |  |
|  | Difference: **27 more per 10000**  (95% UI 7 to 48 more) | |  |  |  |

UI, uncertainty interval.

# **Appendix 16**. Sensitivity analysis for removal of isolation based on a negative antigen test versus ten-day isolation for estimates from relatively simple model for overall patients considering different isolation adherence rates and false negative rates (GRADE summary of findings)

| **Outcome** | **Absolute effect estimates** | | **Certainty of the evidence** | **Plain language summary** |
| --- | --- | --- | --- | --- |
|  | Removal of isolation based on a negative antigen test | Isolation for 10 days |  |  |
| **We assume an isolation adherence of 80% and a false negative rate of 20%** | | | | |
| Onward transmission leading to hospitalisation (28 days) | **200**  per 10000 | **161**  per 10000 | **Very Low**  Due to certainty of parameters (moderate) in the model and indirectness | Whether removing isolation based on a negative antigen test compared with isolation of 10 days would increase onward transmission leading to hospitalisation of secondary cases is very uncertain. |
|  | Difference: **39 more per 10000**  (95% UI 23 to 58 more) | |  |  |
| Onward transmission leading to death (90 days) | **44**  per 10000 | **35**  per 10000 | **Very Low**  Due to certainty of parameters (moderate) in the model and indirectness | Whether removing isolation based on a negative antigen test compared with isolation of 10 days would increase onward transmission leading to death of secondary cases is very uncertain. |
|  | Difference: **9 more per 10000**  (95% UI 2 to 15 more) | |  |  |
| **We assume an isolation adherence of 80% and a false negative rate of 33%** | | | | |
| Onward transmission leading to hospitalisation (28 days) | **234**  per 10000 | **170**  per 10000 | **Very Low**  Due to certainty of parameters (moderate) in the model and indirectness | Whether removing isolation based on a negative antigen test compared with isolation of 10 days would increase onward transmission leading to hospitalisation of secondary cases is very uncertain. |
|  | Difference: **64 more per 10000**  (95% UI 39 to 95 more) | |  |  |
| Onward transmission leading to death (90 days) | **51**  per 10000 | **37**  per 10000 | **Very Low**  Due to certainty of parameters (moderate) in the model and indirectness | Whether removing isolation based on a negative antigen test compared with isolation of 10 days would increase onward transmission leading to death of secondary cases is very uncertain. |
|  | Difference: **14 more per 10000**  (95% UI 4 to 25 more) | |  |  |
| **We assume an isolation adherence of 50% and a false negative rate of 20%** | | | | |
| Onward transmission leading to hospitalisation (28 days) | **274**  per 10000 | **250**  per 10000 | **Very Low**  Due to certainty of parameters (moderate) in the model and indirectness | Whether removing isolation based on a negative antigen test compared with isolation of 10 days would increase onward transmission leading to hospitalisation of secondary cases is very uncertain. |
|  | Difference: **24 more per 10000**  (95% UI 15 to 36 more) | |  |  |
| Onward transmission leading to death (90 days) | **60**  per 10000 | **55**  per 10000 | **Very Low**  Due to certainty of parameters (moderate) in the model and indirectness | Whether removing isolation based on a negative antigen test compared with isolation of 10 days would increase onward transmission leading to death of secondary cases is very uncertain. |
|  | Difference: **5 more per 10000**  (95% UI 1 to 9 more) | |  |  |
| **We assume an isolation adherence of 50% and a false negative rate of 33%** | | | | |
| Onward transmission leading to hospitalisation (28 days) | **295**  per 10000 | **255**  per 10000 | **Very Low**  Due to certainty of parameters (moderate) in the model and indirectness | Whether removing isolation based on a negative antigen test compared with isolation of 10 days would increase onward transmission leading to hospitalisation of secondary cases is very uncertain. |
|  | Difference: **40 more per 10000**  (95% UI 24 to 59 more) | |  |  |
| Onward transmission leading to death (90 days) | **65**  per 10000 | **56**  per 10000 | **Very Low**  Due to certainty of parameters (moderate) in the model and indirectness | Whether removing isolation based on a negative antigen test compared with isolation of 10 days would increase onward transmission leading to death of secondary cases is very uncertain. |
|  | Difference: **9 more per 10000**  (95% UI 2 to 16 more) | |  |  |

# Appendix 17. Sensitivity analysis of estimates from complex microsimulation model for overall patients (GRADE summary of findings)

## 17.1. Sensitivity analysis of five-day isolation versus ten-day isolation for estimates from complex microsimulation model

| **Outcome** | **Absolute effect estimates** | | **Certainty of the evidence** | **Plain language summary** |
| --- | --- | --- | --- | --- |
|  | Isolation for 5 days | Isolation for 10 days |  |  |
| **Use rapid antigen test data for the model** | | | | |
| Onward transmission leading to hospitalisation (28 days) | **197**  per 10000 | **13**  per 10000 | **Very low**  Due to certainty of parameters (moderate) in the model and indirectness | Whether isolation of 5 days compared with 10 days would increase hospitalisation for secondary cases is very uncertain. |
|  | Difference: **184 more per 10000**  (95% UI 183 more to 185 more) | |  |  |
| Onward transmission leading to death (90 days) | **72**  per 10000 | **4**  per 10000 | **Very low**  Due to certainty of parameters (moderate) in the model and indirectness | Whether isolation of 5 days compared with 10 days would increase mortality for secondary cases is very uncertain. |
|  | Difference: **68 more per 10000**  (95% UI 67 more to 68 more) | |  |  |
| **Use viral culture data for the model** | | | | |
| Onward transmission leading to hospitalisation (28 days) | **192**  per 10000 | **8**  per 10000 | **Very low**  Due to certainty of parameters (low) in the model and indirectness | Whether isolation of 5 days compared with 10 days would increase hospitalisation for secondary cases is very uncertain. |
|  | Difference: **184 more per 10000**  (95% UI 183 more to 184 more) | |  |  |
| Onward transmission leading to death (90 days) | **67**  per 10000 | **4**  per 10000 | **Very low**  Due to certainty of parameters (low) in the model and indirectness | Whether isolation of 5 days compared with 10 days would increase mortality for secondary cases is very uncertain. |
|  | Difference: **63 more per 10000**  (95% UI 63 more to 64 more) | |  |  |

UI, uncertainty interval. Model assumptions: we assumed the isolation adherence is 100% and in the daily rapid testing strategy, the test adherence is 100%.

## 17.2. Sensitivity analysis of removal of isolation based on a negative antigen test versus ten-day isolation for estimates from complex microsimulation model

| **Outcome** | **Absolute effect estimates** | | **Certainty of the evidence** | **Plain language summary** |
| --- | --- | --- | --- | --- |
|  | Removal of isolation based on a negative antigen test | Isolation for 10 days |  |  |
| Onward transmission leading to hospitalisation (28 days) | **67**  per 10000 | **13**  per 10000 | **Very low**  Due to certainty of parameters (low) in the model and indirectness | Whether removing isolation based on a negative antigen test compared with isolation of 10 days would increase hospitalisation for secondary cases is very uncertain. |
|  | Difference: 54 **more per 10000**  (95% UI 53 more to 54 more) | |  |  |
| Onward transmission leading to death (90 days) | **27**  per 10000 | **4**  per 10000 | **Very low**  Due to certainty of parameters (low) in the model and indirectness | Whether removing isolation based on a negative antigen test compared with isolation of 10 days would increase mortality for secondary cases is very uncertain. |
|  | Difference: **23 more per 10000**  (95% UI 22 more to 23 more) | |  |  |

UI, uncertainty interval. Model assumptions: we assumed the isolation adherence is 100% and in the daily rapid testing strategy, the test adherence is 100%.
